# The Honolulu Liver Disease Cluster at the Medical Center: Its Mysteries and Challenges

**DOI:** 10.3390/ijms17040476

**Published:** 2016-03-31

**Authors:** Rolf Teschke, Axel Eickhoff

**Affiliations:** Department of Internal Medicine II, Division of Gastroenterology and Hepatology, Klinikum Hanau, Teaching Hospital of the Medical Faculty, Goethe University Frankfurt/Main, Leimenstrasse 20, D-63450 Hanau, Germany; Axel_Eickhoff@klinikum-hanau.de

**Keywords:** Centers for Disease Control and Prevention, Food and Drug Administration, Hawaii Department of Health, Honolulu Queen’s Medical Center, OxyELITE Pro, acetaminophen

## Abstract

In 2013, physicians at the Honolulu Queen’s Medical Center (QMC) noticed that seven liver disease patients reported the use of OxyELITE Pro (OEP), a widely consumed dietary supplement (DS). Assuming a temporal association between OEP use and disease, they argued that OEP was the cause of this mysterious cluster. Subsequent reexamination, however, has revealed that this QMC cohort is heterogeneous and not a cluster with a single agent causing a single disease. It is heterogeneous because patients used multiple DS’s and drugs and because patients appeared to have suffered from multiple liver diseases: liver cirrhosis, liver failure by acetaminophen, hepatotoxicity by non-steroidal antiinflammatory drugs (NSAIDs), resolving acute viral hepatitis by hepatitis B virus (HBV), herpes simplex virus (HSV), and varicella zoster virus (VZV), and suspected hepatitis E virus (HEV). Failing to exclude these confounders and to consider more viable diagnoses, the QMC physicians may have missed specific treatment options in some of their patients. The QMC physicians unjustifiably upgraded their Roussel Uclaf Causality Assessment Method (RUCAM) causality scores so that all patients would appear to be “probable” for OEP. However, subsequent RUCAM reassessments by our group demonstrated a lack of causality for OEP in the evaluated QMC cases. The QMC’s questionable approaches explain the extraordinary accumulation of suspected OEP cases at the QMC in Hawaii as single place, whereas similar cohorts were not published by any larger US liver center, substantiating that the problem is with the QMC. In this review article, we present and discuss new case data and critically evaluate upcoming developments of problematic regulatory assessments by the US Centers for Disease Control and Prevention (CDC), the Hawaii Department of Health (HDOH), and the Food and Drug Administration (FDA), as based on invalid QMC conclusions, clarifying now also basic facts and facilitating constructive discussions.

## 1. Introduction

Hawaii is an exciting archipelago, consisting of eight major and numerous smaller islands, that is famous for its recreational potentials. All are volcanic in origin; each island is made up of at least one primary volcano, although many islands are composites of more than one. Because of their established cultural traditions and nature-based phenomena and secrets, the Hawaiian islands are riddled with ancient mysteries, myths, and stories handed down from one generation to the other [1,2]. In 2013, similar tales, secrets, and mysteries were presented: this time, in Honolulu, by the Queen’s Medical Center (QMC), with its liver disease cluster. This reported cluster, which emerged at Honolulu’s Liver Transplant Center (LTC), created interest but also considerable controversies [3,4,5,6,7,8,9]. The cluster and the seven founding cases were also the basis for statements and conclusions provided by the US Centers for Disease Control and Prevention (CDC), Food and Drug Administration (FDA), and the Hawaii Department of Health (HDOH), subsequently summarized in short as “regulators”.

Disease clusters are challenges in clinical and epidemiological medicine. A cluster refers to an unusual aggregation of health events that are grouped together in time and space. Nowadays, clusters are usually reported to public health agencies [10,11]. Well-known examples of clusters include the epidemic of cholera in London around the year 1850 and the investigations of legionnaires with pneumonia at the Bellevue Stratford Hotel in Philadelphia in 1976. Other notable examples of disease clusters are angiosarcoma among vinyl chloride workers and phocomelia associated with the use of thalidomide. In each of these clusters, one single agent caused one single disease.

Clusters of cases of liver diseases are well described in the literature, in both underdeveloped and developed countries. Since the Honolulu liver diseases occurred in the summertime, were clustered geographically, and were localized to Hawaii, infectious and toxic causes are the first causes that should have been considered. These include infections by hepatitis A virus (HAV) [12,13], hepatitis E virus (HEV) [14,15,16,17,18,19,20,21,22,23,24,25], or leptospirosis [26,27]. Other hepatotoxic agents that should also have been considered include aflatoxins [28,29,30,31,32,33,34,35,36], unsaturated pyrrolizidine alkaloids (PAs) [37,38,39,40], green tea extracts [41], noni juice [42], and kava [43,44,45,46,47,48], just to name a few examples [9]. In addition, tropical diseases were not considered as tentative causes for the liver patients at QMC in any of the reports [3,4,5,6,7,8,9]. Dengue fever is a virus infection transmitted to humans by the bite of a mosquito infected by one of the four different types of the viruses. It is well known that Hawaii was hit by sporadic Dengue outbreaks as local clusters within the past years. As of 13 January 2016, for instance, the current tally of Dengue cases on Hawaii’s Big Island is now at 215, with 195 Hawaii island residents and 20 visitors to the island, having become infected.

However, the QMC’s initial claim of a liver disease cluster by a suspected single dietary supplement (DS) [4] could not be substantiated [6,9]. It soon became clear that the reported group of patients at the QMC did not represent one single cause and one single disease. Instead, the cohort patients used various products and had various diseases and symptoms [6,9]. They suffered not from a single disease but from a variety of liver diseases, and most patients had a significant past history of prior diseases. More importantly, the QMC patients were not exposed to a single product but used a plethora of synthetic drugs with a high hepatotoxic potential, and most also consumed not a single DS but co-used multiple DS’s [6,9]. QMC physicians did not include all of these confounders in their clinical report, presenting incomplete and inconsistent data that were published after a substantial delay since first clinical impressions [5].

A simple case aggregation as a cohort was described for the QMC cases [5]. Such case aggregation or, indeed, any other cohort was not published by other, much larger liver centers from much larger continental US states. It rather appears that the issue of the mysterious case aggregation is primarily a problem that resides at one single place, the QMC in Honolulu [6,9].

In this article, we present new case data and discuss actual upcoming developments in this still highly controversial and challenging issue in more detail to clarify basic facts and facilitate further discussions. Additional consideration is given to key questions how a clinical suspicion based on fragile data of liver patients at the QMC developed to mysteries of invalid clinical and regulatory claims of causality following unjustified upgrading of Roussel Uclaf Causality Assessment Method (RUCAM) causality levels. Intentional upgrading of individual RUCAM scores is a serious clinical, scientific, and regulatory problem, representing an unusual approach in a setting of clinical hepatology aiming to assess correct causality gradings that are reproducible and provided in publications in a transparent form. In fact, RUCAM is user-friendly, and its items are well and clearly defined and allow a robust individual item scoring with the intention to provide a final RUCAM score with various different levels of causality. Finally, we will analyze how these obvious mysteries became a problem of validity, primarily for the medical center and the various regulatory agencies that utilized their dispute findings and fragile conclusions.

## 2. Evolution of the Honolulu Queen’s Medical Center (QMC) Mysteries

### 2.1. Initial Statements and Inconsistencies

The QMC mysteries focus on the question whether OxyELITE Pro (OEP), a popular DS in Hawaii, the continental US, and worldwide, may have caused the QMC liver disease cohort [3,4,5,6,7,8,9]. In the summertime of 2013, these patients were observed by QMC physicians at a single place—their Honolulu QMC [3], which should have caused them to doubt whether a dietary supplement was the cause [4,5,6,7,8,9]. Instead, the QMC physicians claimed a causal relationship between OEP use and liver disease at the QMC [3]. Rather than substantiating their claim in time, they delayed publication of their clinical data and conclusions [5]. Intrigued by these reports, our group analyzed the data and discovered alternative diagnoses for the QMC cases (Table 1) [6,9], replacing previous unsupported diagnoses published by the QMC physicians [5]. The data reported by QMC were selective, ignored overt alternative causes [5,6,9], and had received an intentional and unjustified causality upgrading prior to publication [6,9].

The first official communication of the QMC cases was the preliminary CDC report published on 11 October 2013. This report described the early developments of this mystery of suspected hepatotoxic reactions in connection with the assumed use of OEP in seven patients at the QMC [3]. Entitled “Acute hepatitis and liver failure following the use of a dietary supplement intended for weight loss and muscle building—May–October 2013”, the report implied that OEP was the suspected product, which caused the liver injury, though it also stated that the cases were of unknown etiology.

Since the CDC report was not co-authored by QMC physicians, essential clinical details were not included [3] and open to speculation for an unusually long time [4,5,6,7,8,9]. Therefore, key questions remained unsettled, among these were: why the diagnosis was suspected in the QMC, which algorithm was used to assess causality, and what level of probability in the context of causality was achieved [3,4]. Interestingly, the clinical report of the QMC physicians [5] did not quote or discuss the preliminary CDC report [3], nor were CDC members included as co-authors [5]. It therefore remained unclear to what extent case data and interpretations were exchanged between CDC and QMC, and whether there was agreement on basic issues between the two assessing and reporting parties [3,5].

Overt clinical issues were not included in a Perspective article published in the New England Journal of Medicine on 3 April 2014, again not co-authored by QMC physicians [4]. This article provoked interest and controversy when it reported that epidemiologists at the CDC had confirmed what a liver-transplant surgeon in Honolulu had suspected: OEP was responsible for a cluster of liver cases in the summer of 2013 and had been withdrawn from the shelves [4]. However, the article did not detail how the CDC confirmed the clinical suspicion of the QMC physicians, nor did it define or discuss clear characteristics of the case cohort that make it a cluster [4]. Questions emerged also about the quality of case assessment by the QMC [5,6,9], and which of the various OEP products were used (Table 2) [6,9].

### 2.2. Incriminated Dietary Supplements

The CDC report and the Perspective article did not disclose which of the two popular versions of OEP (Table 2) was the alleged culprit [3,4]. Actually, when the liver cases emerged at the QMC in 2013, OEP with aegeline as one of its ingredients was on the market, later specified as the new OEP types I–III (Table 2) [9]. These replaced a previous OEP product, called the old OEP, with DMAA (1,3-dimethylamylamine HCl) rather than aegeline as one of its ingredients (Table 2) [9]. Regulators and clinicians did not make clear why DMAA or aegeline should be hepatotoxic [3,4,5,6,7,8,9]; among others, DMAA and aegeline were also clearly labelled as product ingredients [9].

It appears that both reports considered the two OEP product groups globally and interchangeably [3,4]. The ambiguity over the identity of the supplement retarded further scientific, regulatory, and clinical assessments and is likely to contribute to inconsistent conclusions. In future cases of possible clusters, clear product identification is mandatory and crucial.

### 2.3. Case and Product Analyses

On 11 October 2013, the CDC report mentions that results from FDA product testing were pending [3]. In late 2015, after a delay of more than two years, regulators provided information that indicates that the tested OEP samples appeared to contain only those ingredients listed in the product information, and at levels indicated [8]. In other words, the products were not with any undisclosed ingredients. Furthermore, in October 2013, FDA informed the distributor of OEP that the agency concluded that aegeline was a new dietary ingredient for which a notification was required, and that the distributor had failed to inform the FDA of the basis for concluding that a DS containing this new dietary ingredient would reasonably be expected to be safe. Ingredients of the various OEP products are listed (Table 2).

### 2.4. Selected Clinical Issues Confronting the QMC

QMC patients used herbal products as well as synthetic drugs, which requires assessment of herb induced liver injury (HILI) as well as drug induced liver injury (DILI) [6,9]. Both are complicated diagnoses that call for a systematic case evaluation, including causality assessment since diagnostic biomarkers commonly are not available for most types of these injury cases [49,50,51,52,53,54,55,56,57,58]. Clinical features, laboratory results, imaging data, or liver biopsy results alone are unspecific and rarely of diagnostic value [53]. Additionally, confounding variables are commonly observed in HILI and DILI and may impede clinical and causality assessment [24,25,50,54,56]. The selective information, which was published by the QMC [5] and others [3,4] about the mysterious liver disease cases at the QMC, raises special concerns [3,4,5]. Instead of the type of information the QMC provided [5], cases of suspected HILI and DILI should be well documented [58] and investigated according to a stringent conceptual approach. 

Essentials for a valid assessment of a liver case cohort are summarized as a ten-point plan, whereby physicians must ensure and verify the following specific requirements: (1) Clear case definition with mandatory criteria; (2) Causality was carefully verified and exists between product use and liver disease; (3) Causality assessment considered confounding variables such as preexisting and coexisting liver diseases, missing clinical data, polymedication, and criteria of challenge and dechallenge; (4) Incriminated product was clearly identified and consistently documented in the records, substantiated by proof of product purchase and use; (5) Clinical assessment was prospective and based on a valid diagnostic protocol; (6) Alternative causes were validly excluded according to accepted guidelines; (7) Treatable diseases were searched for and appropriate therapy was provided; (8) Involved physicians are sufficiently qualified and the corresponding author of a tentative publication has the board examination in gastroenterology/hepatology; (9) Superiors are personally engaged in clinical care of the patients, controlled the patients’ charts and approved the presented clinical conclusions; (10) Medical charts approved by the superiors are provided as raw data to regulators for final approval and initiation of regulatory steps if needed.

This stepwise approach considers most of the typical features of HILI and DILI cases. If consequently applied by the QMC physicians, it could have prevented the mysteries at the QMC. For clarity and transparency, we present not only final and alternative diagnoses for the QMC patients (Table 1) but also their narratives (Table 3) [6,9].

### 2.5. Temporal versus Causal Association

It is unclear on what grounds the QMC physicians claimed the validity of their diagnosis because they considered primarily a temporal association as sufficient evidence of a causal association, describing OEP as the sole causative agent for their mysterious liver disease cases at the QMC. However, valid causation requires a stepwise diagnostic approach [6,9]. We have serious concerns about whether the QMC used a diagnostic protocol stringent enough to validly exclude alternative diagnoses, or rather any protocol at all, because information about any such protocol is missing [3,4,5].

### 2.6. Retrospective versus Prospective Analysis

The QMC physicians did not undertake a prospective approach for analysing their liver cases; at least not such approach was mentioned in public statements by them and others [3,4,5]. Instead, the QMC analyzed cases only retrospectively [5]. Such retrospective analysis has the distinct disadvantage of using case data that are mostly incomplete and unsuitable for a robust case evaluation. Careful prospective data collection of suspected cases [58] can help ensure that alternative diagnoses are not overlooked [50,59] and that the correct diagnoses are found [53].

### 2.7. Quality versus Quantity

The information published by the QMC and regulators about the Hawaii cases showed a distinct preference for case quantity over data quality. They obtained high numbers of cases and seemed to have disregarded the quality aspects of case data and evaluation—to bolster their initial claims and conclusions [3,4,5]. The published conclusions derived from work of the QMC gave few details of only low quality data [3,4]. Instead, it is better if clinicians and regulators focus on a few well-documented cases with high quality data, which would allow them to make a firm causality attribution. Many cases with few, incomplete, and contradictory details inevitably lead to inconsistent conclusions [3,4,5,7,8]. These uncertainties call for a careful analysis of all published cases [3,4,5,6,7,8,9]. 

Two early reports were risky by publishing high numbers of obviously poor-quality cases without a valid causality assessment [3,4]; this approach put the QMC physicians under additional pressure to collect as many cases as possible, independent of their data quality. The risk of case duplication and incorrect case counting now rises with the number of publications, since each report presents different numbers of cases, which are of dubious identity and do not allow a simple addition [3,4,5,6,7,8,9]. Resulting inconsistent and contradictory case data thus creates additional impediments to careful analysis.

### 2.8. Newspaper and TV Publicity Obscuring Careful Clinical Work

The initial claim that OEP caused liver illnesses in Hawaii [3,4] foreclosed a subsequent careful, unbiased case assessment by the QMC physicians [5]. The unexpectedly high local and nationwide publicity, in newspapers and on TV, was initiated, promoted, or at least tolerated by the QMC. This provided a large list of press releases, two of which are cited as examples [60,61]. In addition, regulators provided many press releases online to inform the public, consumers, and physicians of the potential health risk. Lacking proportion, clinical scrutiny, and scientific judgment, this extraordinary publicity put assessors at the QMC as well as regulators who reported the cluster under enormous pressure to succeed, risking biased evaluations and hasty conclusions [3,5,6,9,60,61]. This intense publicity on an unsettled, heavily disputed, and complex clinical topic is indeed unusual in the scientific community; the time pressure forces errors and premature, unjustified conclusions. Such a situation is risky for regulators, physicians, and patients [5,6,9]. Intense publicity also ensures a high reporting rate of suspected cases of low data quality, amplifying the issue of quality *versus* quantity.

### 2.9. Remarkably High Use of Dietary Supplements

Around half or more of adults in the USA report currently using at least one DS [62,63,64]. These impressive figures are supported by a study of researchers from the University of Hawaii in Honolulu carried out in populations residing in Hawaii and Los Angeles [64]. Based on these estimates, it is reasonable to assume that about half of the Hawaiian patients with liver diseases presenting to the QMC in 2013 had reported prior use of at least one DS. The QMC should have assessed all these DS’s individually. Instead, the QMC did not consider and thoroughly evaluate other DS’s [3,4,5] or other possible causes, focusing instead on OEP [3]. Indeed, the list of DS’s consumed by the mysterious QMC cohort is remarkably long (Table 4) [6,9]. Virtually all of these DS’s used by the QMC patients (Table 5) remained unpublished and unassessed [5] as possible causes and confounders for the QMC cases [6,9]. The complete list of these additionally consumed DS’s was not published in the clinical QMC report [5].

The QMC physicians should have established and evaluated an appropriate control group of patients. These would be patients with liver disease but who used other DS’s and not OEP. The QMC could have focused on the final diagnoses of the liver diseases in this cohort. Such case-control studies are standard practice. It is difficult to reconcile with scientific norms this dismissal of a control group that is urgently needed under complex and disputed conditions like these.

## 3. Inconsistencies during QMC Case Analyses

In any case of suspected adverse reaction by a product, there is only one pragmatic approach that merits further consideration. Physicians must be certain and evidence must be provided that the product was in fact consumed. If uncertainty exists about this point, it is not warranted to attempt to attribute causation. Such an assessment would be a waste of financial and human resources. Conducting a causality assessment under these conditions of uncertain product use may lead to invalid conclusions, unjustified accusations, and unwarranted claims. QMC physicians, however, did not implement this pragmatic approach [5]. The worst-case scenario is the combination of both poor causality assessment and poor product identification, with known results of overall declined causality attribution for OEP [6,9].

### 3.1. Product Identification Including Proof of Purchase and Usage

QMC physicians did not make clear how they verified whether their patients actually purchased OEP, which version the patients purchased, and how they used it (Table 1, Table 2, Table 3 and Table 4) [5]. A lack of product identification in cases of suspected herbal hepatotoxicity is a well-known problem, early recognized by the US Pharmacopeia [65] and confirmed as confounder when those cases were reevaluated [66]. For the US Pharmacopeia cases [65], shortcomings of both causality assessments and product identification led to the conclusion that a causal relationship was lacking between assumed product use and liver disease [66], conditions resembling the QMC case mystery [5,6,9] with its lack of causality [6,9]. The statements by the QMC and regulators also merit further consideration, since regulators should also have confirmed exposure to a product, including through proof of purchase [3]. In the QMC cases, proof of product use and purchase was not done [3,4,5] and only rarely reported due to insufficient data documented in the files [6,9]. Aspects of verifying product purchase and use was not considered or discussed as confounders in the two recent reports, undermining their conclusions [7,8].

In the medical records of some QMC patients, OEP use was inconsistently documented at various time points during the hospital stay [6,9]. It appears that use of OEP was often only reported after OEP was highly publicized in newspapers and on TV, or after QMC physicians had questioned patients about whether they used OEP. Overall analysis of the records leaves the impression that at least some of the interviews might have biased patients to report use of OEP. Notes of requests for proof of OEP purchase were not found in the medical records. For one patient (case 2) (Table 3 and Table 4), it was promised that the used bottle will be provided, but this promise was not fulfilled, or at least not documented in the files [9]. According to the records, this patient (case 2) “admitted” OEP use four days after liver transplantation, and had previously refuted use of any DS when admitted to the hospital and on subsequent days, despite multiple questionings. Curiously, multiple drugs for migraine and an OEP bottle were found in the car of this patient, as claimed by her significant other; however, product bottles were not provided [9]. In another case, Oxy Cleanse (OC) use was initially documented in the files, and it is not clear whether and how this was confused with OxyELITE Pro (case 7) (Table 3 and Table 4). For most of the cases, issues of product identification and use are now a court matter. However, even assuming that OEP was consumed by the patients [5], causality still cannot be established [6,9].

### 3.2. Question of Therapeutic Options other than Liver Transplantation

When a product is suspected to have caused liver injury, the first step is to ensure that patients stop taking it. However, it is often difficult to determine whether the injury is related to a preexisting liver disease with specific therapeutic options, and missed alternative causes in suspected DILI and HILI cases are common [50,59,66]. For the QMC cases, lack of a thorough search for treatable liver diseases may have led to further harm to the patients (Table 1) [6,9]. In clinical practice, doctors must follow standard protocols for assessing and treating liver diseases. It is essential that the correct diagnosis be discovered early, so that the disease can be treated with the appropriate therapy, preventing the illness from worsening. Otherwise, if the correct therapy is not used, illness can become severe and fatal, requiring a liver transplantation; acute illness can also become chronic. Therapeutic decisions should be made on a case-by-case basis, also adjusting the dosage to the severity of the disease. Therapeutic efficacy is greater if the liver disease is caught early enough. Clearly, not all treatments are successful, especially in preterminal stages of the liver disease.

All published reports focused on assumed hepatotoxicity by OEP. None reported on alternative diagnoses, and none stated that a specific therapy was discussed or initiated [2,3,4,5,7,8]. We discuss below some treatable liver diseases as few examples with reference to the QMC cases (Table 1) [6,9]. This should serve as a reminder for physicians in care of similar cases that other causes of liver disease should be considered.

#### 3.2.1. Acetaminophen

Although well-known in mainstream medicine for its significant hepatotoxic potential [67,68,69,70,71,72,73,74,75,76], acetaminophen (AAP), or its synonym paracetamol, was not considered a potential cause of the QMC liver cases in Hawaii (Table 1) [6,9], not by the QMC physicians [5], and not by the CDC epidemiologists [3]. This represents a significant flaw in the investigations by both the physicians and regulators [3,5,6,9], considering the available effective therapeutic options by early oral or intravenous application of *N*-acetylcysteine (NAC) to supplement hepatic glutathione stores [67].

AAP was suggested and discussed as a cause in some of the QMC cases. It is a much more likely alternative to OEP, for which causality could not be established [5], as outlined by our group [6,9]. In our reports, we evaluated the medical and other records of five QMC patients and found that all patients had a documented AAP use, but AAP quantification was not routinely done by the QMC. In particular, a procedural diagnostic protocol to assess AAP levels in the blood in all patients at admission was not documented in the files. A few toxscreens were done in the further course but these were likely too late, allowing any AAP to disappear from the blood due to hepatic metabolism [6,9]. The high ALT values of the QMC cases [5,6,9] are in line with those reported for AAP-associated hepatotoxicity and acute liver failure (ALF) [70]. In one of these patients (case 2), who later required a liver transplant [5], her liver specimen obtained by liver biopsy was assessed by a pathologist, as documented in the medical records [9]. He provided details of the liver histology and specified that consideration of AAP toxicity is warranted given the patient’s history of gastric sleeve/gastric bypass procedure, and he even opined that this is a known risk factor for such toxicity [9]. For unknown reasons, this recommendation was not implemented by the QMC physicians, remained without commentary in the medical records [9], and was not included in the published histology description [5]. NAC therapy was not offered to this patient (case 2) (Table 1) [9] during her hospital stay prior to liver transplantation. This transplantation may have been prevented by simple NAC administration [9]. Since AAP was not considered or proven as a cause in any of the other QMC liver patients (Table 1), NAC therapy was not specifically provided [3,5,6,9]. These were poor basic conditions for QMC patients seeking medical help for their liver diseases.

Though the QMC and regulators said that all patients were previously healthy before their liver illnesses [3,5], the QMC patients are best described by their multimorbidities [6,9]. Some suffered from multipain syndromes, most had polymedication, and all of the assessed patients had used AAP [6,9]. Patients susceptible for AAP hepatotoxicity include those with chronic pain, depression, and substance abuse, including alcohol [70]. Multimedication is a known risk factor for DILI [77] and may also be a risk factor for hepatotoxicity by AAP in the QMC cases [6,9]. Alcohol use as another risk factor for AAP hepatotoxicity is described in some patients [67,68,69,70,71,72,73,74,75,76], early recognized in experimental studies [77]. Alcohol use is also a potential risk factor for AAP hepatotoxicity in one of the QMC patients (case 1) (Table 1) [9].

The CDC reported in a previous study on population-based surveillance for acute liver injury in eight counties comprising Metropolitan Atlanta that AAP-related ALF has become the most common etiology of ALF reported from liver transplant centers in the United States [3], with almost half being unintentional overdose [71]. This AAP specific expertise was certainly not included in the recent CDC report published five years later on the QMC cases at the QMC transplant center in Hawaii [3]. CDC also estimated the ALF incidence in a range of 3.5 and 31.2 cases per million each year in the United States [71]. When applied to the Hawaiian population of around 1.4 million, the incidence estimate equates to around 5 to 44 ALF cases, half of these would likely be associated with AAP use. AAP is widely used as a prescription drug as well as an OTC medication in the continental United States [70,71]. For Hawaii, the AAP issue obviously was ignored and not addressed by the CDC regulators [3]. It was also not captured and remained unpublished by the QMC physicians in their report [5], further evidence of their selective case data presentation [5]. Conversely, AAP use and possible AAP hepatotoxicity was clearly identified in the QMC cases, with AAP as the potential cause of injury [6,9] or as tentative confounder [8]. However, AAP was again not considered in another CDC study [7]. Therefore, the overall clinical and regulatory approach to AAP hepatotoxicity at the QMC is not in line with these mainstream approaches to AAP.

The position paper of the American Association for the Study of Liver Diseases (AASLD) on the management of acute liver failure provides clear details for necessary diagnostic and therapeutic approaches in its 2011 update [75]. These guidelines include: (1) The precise etiology of ALF should be sought to guide further management decisions; (2) for patients with known or suspected AAP overdose within four hours of presentation, give activated charcoal just prior to starting NAC; (3) begin NAC promptly in all patients where the quantity of AAP ingested, serum drug levels or rising aminotransferases indicate impending or evolving liver injury; (4) NAC may be used in cases of ALF in which AAP ingestion is possible or when knowledge of circumstances surrounding admission is inadequate but aminotransferases suggest AAP poisoning; (5) obtain details (including onset of ingestion, amount and timing of the last dose) concerning all prescription and non-prescription drugs, herbs, and dietary supplement taken over the past year; and (6) NAC may be beneficial for ALF due to DILI. If some of these AASDL recommendations were followed and applied to the QMC patients [75], most of the present clinical QMC dilemma would have been prevented, including the discussion around AAP induced ALF and liver injury in at least two patients [9].

#### 3.2.2. Hepatitis B Virus Infection

The AASLD position paper on ALF states that nucleos(t)ide analogues should be considered for hepatitis B-associated ALF [75]. When analyzing the medical records of the QMC cases for one patient (case 3) (Table 1), our group discovered positive tests for anti- hepatitis B surface (anti-HBs) and anti- hepatitis B core (anti-HBc), suggesting an infection by the hepatitis B virus (HBV) [9]. This HBV infection was somehow overlooked by the QMC physicians and not documented in the files as disease [5]. As a consequence, potential therapy with nucleos(t)ide analogues [75,78,79] was not initiated [9]. Since the patient was not given the appropriate therapy, the clinical course was prolonged, as expected. Recovery was not documented in the files [9] and was not published [5]. Instead, QMC physicians incorrectly attributed a “probable” causality for OEP for this patient [5,9], classified the hepatic encephalopathy with grade 1 [5], and mentioned a severe DILIN grading with a severity grade of 4 [5], although this patient never had DILI but simply a HBV infection [9]. The final MELD was graded as 10, implying severe liver disease caused by OEP [5], ignoring HBV infection as causative [9]. It seems that the QMC physicians did not check their clinical files with the required scrutiny, or did not interpret positive antibody tests of HBV correctly [9]. These clinical issues likely prevented initiation of an effective therapy with the aim of ultimate cure of the HBV infection.

#### 3.2.3. Hepatitis E Virus Infection

Acute HEV infections are normally self-limited diseases with good outcome and without the need of a specific therapy, but patients with severe courses are described who benefit from a specific therapy with ribavirin [14,16,80,81,82]. In 2011, the position paper of the AASLD on the management of acute liver failure correctly stated that ALF related to viral hepatitis E must be treated with supportive care as no virus-specific treatment has proven to be effective [75]. During the past years, however, efficacy of ribavirin therapy has been shown in various reports, and ribavirin therapy should now be discussed on a case-by-case basis and be initiated in patients with clearly established HEV diagnosis and severe clinical course or imminent ALF. Regarding the QMC cases, potential HEV infections and therapy options are major issues in the case discussions since clinical courses were severe and HEV infections are to be considered albeit still a matter of debate [6,9].

Hepatitis of initially unspecified type was the most commonly documented initial diagnosis for the QMC patients at admission, based on clinical features and supporting laboratory data [6,9]. The subsequent clinical course and laboratory results were compatible with or suggestive of HEV infections in some of the QMC patients [6,9]. Regulatory and clinical HEV diagnostic work-up was not described or not handled appropriately and did not allow disproof or proof of HEV as diagnosis [3,5,6,9]. All QMC patients had high alanine aminotransferase (ALT) levels, exceeding 1.000 U/L [5,6,9] and suggesting either AAP or alternatively HEV as cause. As discussed earlier in detail, such high ALT values are compatible with HEV infections [9]. For instance, ALT values of 1970 U/L were suggestive of an acute infection by a hepatitis virus such as HEV in one patient (case 1) (Table 1) [9]. This unusually high ALT value certainly is not in line with DILI by OEP as assumed before [3,5]. In fact, a comparative study of a DILI and HEV cohort published mean serum ALT values of 398 ± 442 U/L (SD) for established idiosyncratic DILI, as opposed to mean serum ALT values of 1410 ± 799 U/L (SD) in patients with proven acute HEV [25]. Not recognized or discussed before [3,5], these differences in liver enzyme pattern may dissociate DILI from HEV on laboratory grounds only [9]. Although two publications dealt with the QMC cases [3,5], it remains unclear why the HEV issue was not adequately handled.

It turned out that Hawaiians are confronted with the HEV issue in two ways: One is related to HEV reservoirs in infected animals in Hawaii, and the other one focuses on problems to correctly identify HEV infections in Hawaiian hospitals [9]. HEV reservoirs in infected animals in Hawaii have been well described [23]. Consequently, exposed persons are prone to acquire HEV infections, a possibility, especially in summertime cluster cases, which should have been discussed in the original reports [3,5] but was detailed only later by our group [6,9]. In fact, attempts to verify or disprove HEV infections in the cluster patients were not discussed or published by regulators [3] or clinicians [5].

After analysis of five Honolulu QMC cases [9], which comprised four cases [9] and a fifth case published earlier [6], it was found that, in two out of these five analyzed cases (40%), neither anti-HEV IgM, anti-HEV IgG, nor HEV PCR equivalent to HEV RNA was assessed, namely, in case 4 [9] and the single case published earlier [6]. In none of the remaining three patients (cases 1–3) was HEV PCR assessed [9]. Anti-HEV IgM was negative in cases 1–3, and anti-HEV IgG was negative in case 2; it was not tested in the others [9]. Although various diagnostic tools for HEV are on the market, the detection of HEV RNA in biologic specimen of serum and/or stools is considered the “gold standard” for the confirmation of acute HEV infection [82]. Failing to perform HEV PCR testing to quantify virus RNA copies [14,82] for all five assessed patients is a highly significant regulatory and clinical problem [3,5,6,9], as it prevents a thorough evaluation of this summertime cluster by ignoring a potential and important alternative cause [3,5]. The patients’ files do not reveal a stringent and transparent regulatory or clinical protocol to resolve the HEV issue in the QMC patients. It appears that HEV antibody tests were ordered only haphazardly. Anti-HEV IgM analyses, which have only a short diagnostic window [14], were sometimes performed during the late phase of liver illness when HEV IgM antibodies may have already vanished.

Most problematic for patients in Hawaii and the continental Unites States, US HEV antibody tests lack FDA approval [14,24] and are plagued by poor sensitivity and specificity [14]. It is unclear whether these test characteristics are related to the various HEV genotypes [14,15,16], raising questions about whether all relevant HEV genotypes are detected by the available HEV antibody tests [9]. For one patient, his files identified a kit from Focus Diagnostics used for anti-HEV IgM testing [83]. Since all patients were cared for at a single medical center, this kit was likely used in all other patients, although not approved by the FDA [9]. The manufacturer recommends that its hepatitis E antibody tests IgM, IgG, and IgM + IgG should not be used for diagnosis without confirmation by other medically established means and declares that these hepatitis E antibody tests have not been cleared or approved by the FDA [83]. Consequently, all HEV antibody test results in the QMC cases are insecure and should be questioned [9].

In other countries like the United Kingdom and Germany, validated HEV antibody tests are approved and marketed [25,81]. These antibody tests should be used in the Hawaii cluster patients to retrospectively test retained patients’ biological material of blood, obtained at various time intervals to investigate HEV infections; in addition, PCR analyses should be done in retained blood, stool, and liver specimens. Actual testing is also essential in the patient with the clinically suspected HEV infection (case 1) (Table 1) who still has increased liver tests (LTs) following orthotopic liver transplantation (OLT), which might be ascribed to rejection events or still ongoing HEV infection episodes [9]. In another patient (case 4), the liver pathologist recommended that testing for HEV be considered, but tests for HEV were not done [9]. Failing to diagnose HEV may result in delaying or withholding an effective therapy in severe HEV infections beyond the point of self-limitation and complete cure and may further harm the affected patient.

#### 3.2.4. Herpes Simplex Virus Infection

For acute hepatitis by herpes simplex virus (HSV) infections, therapeutic options are available with aciclovir [84,85,86,87,88,89]. In more detail, the AASLD position paper on the management of ALF states in its guidelines that patients with known or suspected HSV as the cause of ALF should be treated with aciclovir (5–10 mg/kg intravenously every 8 h) and may be considered for liver transplantation [75]. Importantly, early treatment is recommended even in suspected cases. HSV hepatitis in one patient (case 4) (Table 1) [9] was not published or discussed by the CDC regulators [3] or the QMC physicians [5], although this diagnosis was easily established after thorough analysis of case data [9]. HSV infection as confounder in this patient (case 4) (Table 1) [9] invalidates the regulatory and clinical diagnosis of OEP hepatotoxicity [3,5,9]. This patient was lucky since she received a prolonged therapy with high doses aciclovir for her genital herpes [9]. Genital herpes is a known risk factor for fulminant courses of HSV hepatitis, but the diagnosis can only be established by a physician who is familiar with details of this infectious disease [89]. In another study, a patient with fulminant and initially not recognized HSV hepatitis, HILI was primarily suspected and alternatively also autoimmune hepatitis (AIH) treated by steroids with a fatal clinical course requiring liver transplantation [89]. In fact, hepatitis by HSV infection was established as primary diagnosis after assessment by our group [89], ignored, and not recognized by the US Pharmacopeia [65]. This diagnosis was missed since US Pharmacopeia used the heavily disputed Naranjo scale for causality assessment [90].

Acute HSV hepatitis is usually fatal if this diagnosis and the aciclovir treatment are missed [87]. Among 93 reported patients with HSV hepatitis, 63 had no antiviral therapy, most of them died and only 11% survived. Therefore, in any liver center including the QMC, HSV hepatitis should be considered as a candidate for unknown liver diseases. It remains unclear as to why this diagnosis was not suspected in the QMC patient (case 4) [9] when assessed by clinicians [5] and in the regulatory context [3].

#### 3.2.5. Varicella Zoster Virus Infection

For acute hepatitis by varicella zoster virus (VZV) infections, aciclovir is the treatment of choice [84], with similar recommendations by the AASDL as outlined above for HSV [75]. Among the QMC cases, there was one patient (case 3) in whom our group diagnosed acute hepatitis by VZV (Table 1) [9]. Medical QMC files provided information that this patient was positive for anti-VZV IgM and anti-VZV IgG, substantiating VZV hepatitis as firm diagnosis [9]. With VZV hepatitis as established alternative diagnosis and confounding variable, this makes the diagnosis of OEP hepatitis unlikely [5,9]. It remains unclear as to why QMC and the CDC were not able to capture this diagnosis and to provide the correct therapy.

Experienced clinicians and regulators are certainly aware of the abundant publications that acute VZV may progress to ALF with need for liver transplantation and the high rate of fatal outcome if diagnosis and therapy are missed [75,91,92,93,94]. After cessation of OEP use, recovery was not achieved in the patient (case 3) [5,9]. Such a clinical course is highly suggestive of alternative causes, which were not recognized. The further clinical course of this patient, as well as whether it was self-limited, is unknown. The problem in this patient was that she had actually a coinfection of acute HBV and VZV (Table 1) [9], where both infections remained untreated and undisclosed by QMC clinicians and regulators [3,5], although positive antibody tests were documented in the files and should have allowed an early diagnosis [9]. This case illustrates again the problematic case assessments by QMC clinicians and regulators, and represents an unfortunate burden for the patient.

#### 3.2.6. Cytomegalovirus

Hepatitis by cytomegalovirus (CMV) can be effectively treated by various antiviral drugs including intravenous ganciclovir, although they are mostly self-limited and do not require specific treatments [95,96,97]. However, CMV hepatitis may have a severe or even fatal course [96]. The AASLD position paper does not address the issue of ALF by CMV [75], which should be considered in any case of unclear severe liver disease and ALF. In one of the QMC patients (case 1) (Table 1), anti-CMV IgG was positive but titer changes were not assessed in the further course [9]. This raised the question of whether the patient’s disease may represent resolving acute CMV hepatitis. Overall, some uncertainty exists around CMV diagnosis, whether it may be an infection or a disease, and diagnosis may be complicated considering antibody tests and CMV DNA [96,97]. In this QMC patient, CMV DNA was not assessed in the explanted liver, which could have helped to establish or disprove a diagnosis of CMV hepatitis [5,9].

#### 3.2.7. Wilson Disease

Wilson disease (WD) causes ALF [75,98,99], and it is identified as the primary etiology in around 5% of ALF patients worldwide [98]. This autosomal recessive genetic liver disorder of copper metabolism was not adequately excluded in the QMC cases with rare ALF but mostly severe clinical courses [5,6,9]. Patients with ALF by WD require liver transplantation since chelating therapies are commonly ineffective in this late stage [75,98,99]. However, patients with severe but early stages of WD without ALF profit from effective therapy and commonly need no liver transplantation, provided the diagnosis is early established. The diagnosis of WD in the setting of ALF and earlier stages can be difficult to ascertain, but it is vitally important due to its prognosis, treatment, and implications for family members [98]. In few QMC cases, QMC physicians likely attempted WD exclusion by assessment of ceruloplasmin levels without publishing any details of other mandatory diagnostic approaches [6], because ceruloplasmin as a single parameter is an inappropriate diagnostic measure [75,98,99]. Regarding possible WD, it appears that the QMC patients did not profit from published recommendations [98,99] or the position paper of the AASLD on the management of acute liver failure [75].

To exclude WD, AASDL recommends that one should obtain ceruloplasmin, serum and urinary copper levels, slit lamp examination for Kayser-Fleischer rings, hepatic copper levels when liver biopsy is feasible, and total bilirubin/alkaline phosphatase ratio. Patients in whom WD is the likely cause of ALF must be promptly considered for liver transplantation [75]. However, urine copper levels should be obtained for 24 h as specified [53,98].

### 3.3. Undeclared QMC Case Definition

The tremendous print press and TV publicity [60,61] as well as several publications in scientific journals by authors not affiliated with the QMC [3,4] put pressure on the QMC physicians to publish details on the mysterious liver cases that emerged on their watch. Because QMC clinicians did not define their cases, it was unknown whether the claims they presented were realistic [3,4,60,61]. Only in August 2014, did the QMC physicians finally publish their report in short form, just as a Letter to the Editor [5]. Expectations were high but not fulfilled because QMC physicians did not provide any case definition in their 2014 report or later.

In the meantime, clinical records were analyzed in 2015 by our group. We found no evidence in the files of the QMC patients that a case definition was documented, which is striking given the circumstances [6,9]. At this point, one could argue that the CDC report contained some case definition for their regulatory assessment of the QMC cases, and these definitions might have been used by the QMC physicians [3]. The QMC physicians did not disclose their own case definitions, which they might have used prospectively since May 2013, nor did they refer to the case definition presented by the CDC report [3], which was not referenced by the QMC report [5].

In essence, the scientific community and regulators were not aware until early 2014 whether, and if so which, criteria were used to include or exclude cases in the study cohort at the QMC, thereby resulting in an unjustified claim of hepatotoxic properties of a product allegedly used by a group of the QMC patients. A lack of case definition inevitably leads to questionable conclusions and disputed results [5], as detailed recently after careful analysis of the QMC cases [6,9].

The QMC physicians also did not provide any information how they ascertained product identification, purchase, and use [5]. One could also argue that case criteria are less important than an assumed temporal association between product use and liver disease. However, case analyses were insufficient to establish not only such a temporal association in many QMC cases but also product identification, purchase, and use [6,9]. Therefore, basic requirements for a valid case assessment were impeded.

### 3.4. Incomplete Data Presentation

Honest scientific work requires transparency of all data and does not allow selective data presentation [5] of results that specifically do not support initial claims [6,9]. Actually, there were substantial gaps between documented case data available to the QMC physicians or regulators and data published in the two reports [3,5], as analyzed by our group [6,9]. For instance, it is certainly not a good idea to exclude PMH details when QMC physicians and regulators claim that the QMC patients were healthy prior to their liver illness [3,5], substantiating the criticized presentation of selected case data [3,5,6,9]. Good health condition is not documented in the clinical and other records that show an abundance of prior or concomitant diseases in most of the QMC patients (Table 1, Table 3, and Table 5) [6,9], which was confirmed recently upon regulatory assessment [8]. Evaluation of the PMH of each patient is a clinical cornerstone in mainstream medicine. Actually, some QMC patients, who were documented in the clinical charts as multimorbid, had a multipain syndrome (Table 1, Table 3, and Table 5) [6,9]. Hence, they may be polymedicated also by potentially hepatotoxic synthetic drugs (Table 6) [6,9] with the risk of DILI causing their liver illnesses (Table 1) [6,9]. For all these reasons, careful assessment of prior diseases and drug use is essential [6,9] and should not be disregarded [3,5].

### 3.5. Diagnostic Uncertainty

#### 3.5.1. Qualification

DILI and HILI are highly challenging diseases with different facets that are best diagnosed by experienced hepatologists and gastroenterologists who are board certified. It is unclear whether any such qualified clinician was in charge of caring for the QMC patients whose clinical data were assessed for causality. The documented files raise questions regarding the judgment of the QMC physicians in various sections [6,9]. The best clinical work is done in the absence of internal and external pressure, and when bias is not a problem, but these conditions were obviously not present in the QMC setting outlined above.

#### 3.5.2. Lacking Strict Diagnostic Protocol at the QMC

Analyses of patient and clinical files indicated that a strict diagnostic protocol for case evaluation was missing. Laboratory tests were ordered at the discretion of the physicians, sometimes at the beginning or in the middle of the clinical course, sometimes at the end, or not at all [6,9]. This explains why results of some hepatitis serologies or PCR are available and others are missing (Table 7). A straightforward clinical approach looks different.

### 3.6. Choosing RUCAM as the Best Causality Algorithm

The QMC clinicians used RUCAM for causality assessment [5], which is the best qualified approach compared to other methods [53]. However, instead of applying the updated version as published in 2013 [100], they used the original and outdated version of 1993 [101,102], which they also criticized [5]. Certainly, fairly good results still could have been obtained with the 1993 RUCAM version in 2013 [101,102], but it must be applied correctly [9]. Right now, a new RUCAM version is available to clearly dissociate this version from previous versions that are now considered obsolete [53]. Whatever version is used, assessment should include valid HEV data obtained by FDA-approved antibody tests and be done prospectively to collect all essential and not just selective data [5,6,9].

RUCAM is the ideal causality assessment method for suspected hepatotoxicity by drugs, herbs, and dietary supplements [53,100]. Thus, it is an appropriate method for the QMC patients who consumed various drugs, herbs, and dietary supplements [6,9]. RUCAM is specific to liver illness. It considers all features of hepatotoxicity, is structured, and provides quantitative results based on individually scored key elements that are clearly defined [53,100]. The individual scores of the key elements are summed up and provide a final score with a causality grading: ≤0 points, excluded; 1–2, unlikely; 3–5, possible; 6–8, probable; ≥9, highly probable.

To provide transparency of available or missing data and their individual scoring, RUCAM data should be published as a table—patient by patient, product by product, and item by item [58], as our group has published for the QMC patients [6,9]. Publishing only final RUCAM scores does not allow reevaluation by other scientists, is risky, and raises serious questions about the presented conclusions [58], as shown recently [5,6,9]. Therefore, whenever RUCAM is applied, it must be used carefully and correctly with elements as documented in the files [6,9]. Failure to adhere to these basic prerequisites may easily result in flaws. RUCAM assessments should not be done for products with questionable purchase or use conditions and unclear product identification, because the results will be questionable.

### 3.7. Incorrect Use of RUCAM

#### 3.7.1. Challenge Criteria

Occasionally, the scoring of a RUCAM element remains uncertain for the assessing physician or scientist. In such cases, the item should be marked with a question mark as part of the published item score and final score to clearly indicate the scoring uncertainty. This provisional scoring allows a tentative causality grading, an approach sometimes used by our group [9]. Assessing the QMC cases, scoring problems were evident for a few RUCAM items. For instance, in some cases, the interval between last use of the product and the onset of liver disease was too long and exceeded 14 days [53,100], sometimes with episodes of vomiting and the assurance or assumption that during these times the product was not used any more (Table 3) [6,9]. For some QMC patients, physicians did not appropriately document time frame between the beginning of product use with day 0 as the first day of intake and the onset of increased liver enzymes or symptoms (Table 3) [5,6,9]. Incorrect or inaccurate documentation of challenge characteristics inevitably leads to insufficient dechallenge results and uncertain interpretation. QMC physicians did not publish individual scoring results of their patients, which obscured the causality assessment [5].

#### 3.7.2. Dechallenge Features

Incorrect scoring was also found in the dechallenge domain as assessed by the QMC physicians, since scoring is allowed only when product use was terminated immediately before first ALT values were available but not when termination occurred weeks before [5,6,9]. Dechallenge criteria try to capture and evaluate the natural course of the disease quickly after product use cessation, without any interference by time or any drug therapy that may cause a rapid ALT fall, the therapeutic goal. For the QMC cases, weeks often passed before first ALT values were recorded. Drug therapy usually did not interfere since required drug therapy was withheld due to missing the correct diagnosis (Table 1). In only one patient (case 7), short-term NAC was given, but its effect on ALT dechallenge is not documented (Table 4). Overall dechallenge velocity was slow in the QMC patients, which is highly suggestive of an ongoing liver disease by causes unrelated to the incriminated product (Table 1) [6,9]. In only one patient (case 8), a rapid dechallenge with an ALT fall by more than 50% within eight days was documented and provided +3 points for the new OEP [6]. In half of the four other cases, ALT decline by more than 50% was within 30 day, providing +2 points for the new OEP; in the other half of cases, valid information of ALT decline was missing and provided 0 points [9]. Therefore, ALT dechallenge criteria are cornerstones of the RUCAM evaluation since a maximum of +3 points are potentially achievable, but valid documentation is needed [6,53,58]. The clinical QMC report failed to provide individual dechallenge scores but mentioned prolonged clinical courses not compatible with a quick ALT decline associated with a rapid resolution of the disease and not suggestive of high final scores required for a probable causality level [5].

#### 3.7.3. Comedication Issue

Among the QMC patients, comedication was frequent in some patients with >10 drugs or >17 DS’s (Table 3, Table 4 and Table 6) [6,9] and remained unpublished in the clinical QMC report [5] although well documented in the files, which were recently reassessed [6,9]. Comedication commonly leads to subtraction of scores and a low final score [53,58,101], as was seen in the QMC cases after reevaluation [6,9]. *Vice versa*, hiding comedicated drugs, herbs, and DS’s from evaluation and publication inevitably upgrades the RUCAM scores to higher causality levels that are misleading and unacceptable [5,6,9]. Even more importantly, each individual drug, herb, or DS has to be evaluated separately with the RUCAM scale, with an individual score for each agent. This was not done in the original QMC report [5]. This again is a major procedural flaw that allowed QMC physicians to publish unjustified high RUCAM scores with high causality gradings for their claims that OEP is responsible for liver diseases of their patients [5].

#### 3.7.4. Alternative Cause Search

In this domain, RUCAM considers the clinically most relevant alternative causes and complications of underlying disease(s) [53,56,58,101]. First, it contributes scores for causality assessment [6,9,53,58]; Second, it is a reminder whether the diagnostic work up was complete; regarding the QMC cases, several alternative causes were ignored and never investigated (Table 7); Third and most importantly, the RUCAM analysis disclosed many alternative causes and treatable diseases in the QMC cases (Table 1 and Table 7) [6,9]. These diseases had not been recognized, treated, or published by the QMC physicians before [5]. It is also evident that the QMC physicians did not resolve their uncertainty regarding HEV diagnoses [6,9] and did not discuss this issue in their publication [5].

#### 3.7.5. Willingly Upgrading of RUCAM Scores

The published RUCAM-based analyses erroneously attributed total RUCAM scores of 6–7 for most of their QMC cases [5]. Such high scores commonly are a privilege of cases with excellent and complete data sets but are not achievable with problematic case data like the QMC data [6,9]. Scoring of the QMC cases must be substantially reduced based on the multiple unpublished and ignored potentially hepatotoxic drugs as comedication [5] as well as the incomplete case data and poor dechallenge data [6,9]. The files of the patients also commonly do not document valid ALT decreases by ≥50% within eight days after cessation of drug or DS use and not just after admission, and exclusion of most likely alternative causes was insufficient, which also reduced considerably individual and total scores [6,9]. The incorrect transfer of basic data from the clinical charts and other documents [6,9] into the publication [5] is significant.

We have learnt from our reanalyses of the QMC cases and statements made in court in Honolulu that the QMC physicians had willingly upgraded individual and final RUCAM scores without a clear justification. The results and conclusions published by the QMC physicians are therefore questionable at best [5]. This unlawful upgrading is an extremely annoying situation in clinical medicine and science and leads to unacceptable consequences for physicians, scientists, regulators, and patients.

### 3.8. Diagnosis of Exclusion

DILI and HILI are diagnoses of exclusion in mainstream medicine [14,56,57,58,59,101,103]. Claiming DILI or HILI by OEP requires valid exclusion of alternative causes [6,9]. However, the QMC’s attempts to exclude alternative causes in their patients failed and were not guided by a valid approach because the QMC physicians did not account for many alternative causes (Table 1, Table 3, and Table 7) [6,9]. In particular, QMC physicians were not able to validly exclude HEV in their QMC cases [5] by using correct and approved methods of HEV evaluation (Table 7) [6,9]. Many publications provide clear evidence that HILI and DILI are not injury cases unless HEV is validly excluded [14,24,25,56,57,58,59,101,103,104]; again emphasizing that hepatitis E easily masquerades as a DILI [104].

### 3.9. Critical Liver Histology

The issue of liver histology in the QMC patients was not adequately handled in the QMC publication because it presented four different histology cross-sections without any case attribution [5]. These cross-sections were interpreted as typical OEP hepatotoxicity, a fading diagnosis due to lack of causality [6,9]. Prior to their assessment, pathologists were informed by the QMC physicians about the patient’s OEP use, with the risk of biased conclusions [9]. Recommendations of the pathologists to consider alternative causes such as AAP or HEV were ignored by the QMC physicians [9]. Overall, these histology descriptions are now irrelevant and obsolete [5] since the existence of OEP hepatotoxicity must be denied [6,9].

## 4. Emerging Problems of the QMC Credibility

By erroneously ascribing their liver disease to suspected hepatotoxic properties of OEP and claiming a strong causality, the QMC physicians involved these patients in mysteries that accumulated at their QMC [3,5]. The QMC physicians stated that they had shown causality, a strong word, without providing timely and clear evidence to support this claim [3,5,6,9]. For the US, such case accumulation in Hawaii was unique [3,5], as similar case accumulations were not observed in any other larger medical or liver transplant center of continental US states [7].

Clinical and patient chart analyses of our group revealed inconsistencies between documented and published data as well as unjustified causality upgrading, which led to the QMC’s invalid causality levels and clinical conclusions [6,9]. Increasing the scores of their patients raises questions as to the validity of the QMC physicians’ conclusions. Consequently, we became aware of a chain reaction, which started with unjustified claims of causality, biased case evaluations and publicity, unconsidered major confounders, unrecognized and treatable alternative liver diseases, and uncovered data inconsistencies (Table 1 and Table 7) [6,9]. At the end of this chain reaction, misdiagnoses and problematic data with causality upgradings prevailed and biased case assessments and conclusions by regulators in 2014 and late 2015 [7,8]. Detection of the QMC’s flaws was published only in mid 2015 and early 2016 [6,9]. It seems that the QMC problems expanded to regulators and patients [6,7,8,9]. While QMC’s approach has been determined to be faulty, the scientific community is faced with analyzing how this problem could have happened.

## 5. QMC Challenges

### 5.1. General Considerations

The mystery at the QMC emerged in 2013 [3,4,5,6,7,8,9]. QMC has a LTC with physicians who care for patients with liver diseases, a usual course of events in clinical medicine. Since half of the adults in the continental Unites States use at least one DS [62,63,64], it is likely that around half of the patients with liver disease, who showed up at the QMC in 2013, had a prior use of DS’s. At that time, OEP was a very popular DS, commonly used throughout the US [4]. Putting things together, the QMC physicians hypothesized a claim that seven of their liver patients at the QMC were injured by the use of OEP [3,4]. QMC physicians published their results of formal causality assessments of their cases only in late 2014 [5].

### 5.2. Problems with QMC Data and Confounders

In 2015, case reassessment by our group [6,9] and the physicians’ acknowledgement in court of unjustifiable significant upgrading of RUCAM causality scores in their patients at the QMC inevitably led to the conclusion that the QMC case data of causality gradings were invalid and did not prove a causal relationship between OEP use and liver disease [9], a conclusion also reached in the present article.

The QMC’s case and data management was problematic. Problems included case data retrieving, selection, documentation, interpretation, and public presentation [3,5,6,9]. Significant discrepancies between the published account and the facts documented in the medical records have been summarized [6,9]. Close analysis of the complete clinical data provided insight into how additional or misfitting data were neglected, misinterpreted, or somehow removed from further assessment to support the initial OEP concepts [6,9].

Nowhere is it documented or published that the QMC physicians used any standardized questionnaire to provide a firm basis for their claims, or that a control group at the QMC had been defined and assessed by clinical and regulatory standards, and nowhere has a critical statement been published on confounding variables. Problems, however, dominate the QMC case assessment [5] and are evident in numerous areas following careful analyses, as summarized in listing compilations in recently published papers [6,9]. Confounders are a problem of the QMC publication [5], regulatory reports [3,7,8], and other statements [4]. Some of these confounders are presented as examples (Table 8). 

## 6. Centers of Disease Control and Prevention Report

### 6.1. First Regulatory Report

Except for the media articles, CDC epidemiologists were the first who reported on the mysterious seven cases with liver disease at the QMC of “unknown etiology”, though the actual message was that a causal relationship to the use of OEP was postulated [3]. For this CDC report, QMC physicians were not listed as co-authors and do not appear to have contributed.

### 6.2. Public Health Investigation

CDC reported that on 9 September 2013, the HDOH was notified of the seven patients at the QMC with severe acute hepatitis and fulminant liver failure following OEP use [3]. The HDOH, with the CDC and the FDA, initiated a public health investigation including patient interviews and medical chart reviews, and CDC pointed out that the patients were healthy before [3], which was not substantiated since multimorbidities were uncovered upon reanalysis of case files [6,9].

### 6.3. Epidemiological Study Protocol with Missing Control Group

In the fall of 2013, the epidemiologists employed by the regulatory agencies did not take the opportunity to define and evaluate an appropriate control cohort, which takes into account actual epidemiology criteria [3]. Any case control study done later than the fall of 2013 would be inadequate because the proper data could not be collected [9]. This problem with the epidemiological and clinical aspects of the analysis invalidates the conclusions of their reports [3,5]. A control cohort lacking DS use should have been defined at latest in the fall of 2013—earlier would have been even better—consisting of patients with acute hepatitis or ALF of initially unknown etiology, occurring after 31 March 2013, and in care of the Honolulu QMC.

Patients of this control group should not have reported any DS consumption within the previous 60 days but fulfil the regulatory criteria for hepatotoxicity, e.g., serum ALT >4N and total bilirubin >2N, with a valid diagnosis and established cause of the patients’ liver disease for comparison.

### 6.4. Lack of Specific Product Identification

The specific OEP product cited as the basis for the reported liver cases by the CDC remained obscure [3]. This lack of product identification delays further assessment, since several OEP types were available on the market (Table 2).

### 6.5. Questionable Causality Assessment Method

The CDC did not report which diagnostic algorithm was applied to assess causality in the QMC cases, with the result that causality gradings remained unpublished, thereby expanding problems with the analysis of the cases at the QMC [3].

### 6.6. Inadequate Regulatory Case Definition

Subsequently, the regulators HDOH, CDC, and FDA defined a case as acute hepatitis of unknown etiology, occurring after 31 March 2013, in a person who had consumed a DS within the previous 60 days and had a serum alanine aminotransferase (ALT) four times the upper limit of normal (N) and a total bilirubin level greater than or equal to 2N; negative serologies for infections including viral hepatitis were mandatory [3]. However, this regulatory case definition is imprecise and problematic for various reasons. First, such definition was obviously not used by the QMC physicians for the QMC patients nor was it published in the clinical QMC report [5], which did not even reference this CDC report [3]. Second, such case definition disregards the period from cessation of DS use to disease onset, thus ignoring the important criterion of a strict temporal association; according to mainstream opinion, tentative hepatotoxicity is assumed only if liver disease is manifested within a short time window after the last use of the product, commonly two weeks or less (a maximum of four weeks is sometimes used for slowly metabolized compounds) [101]. Third, hepatotoxicity commonly is no longer considered for ALT values <5N [53,56,57,105], so that the inclusion of patients with unspecific ALT elevations is avoided. Fourth, the agencies did not specify how they excluded preexisting liver diseases, or how they evaluated infections including viral hepatitis. Thus, they did not account for some crucial elements for assessing causality in suspected HILI and DILI [3]. To exclude various viral hepatitis forms, anti-HAV IgM is tested for HAV; anti-HBc IgM and HBV-DNA for HBV; anti-HCV and HCV RNA for hepatitis C virus (HCV); and PCR analyses for viral DNA and titer changes of specific IgM and IgG antibodies for infections by HEV, CMV, Epstein Barr virus (EBV), HSV, and VZV [53,57,58,101]. Fifth, the agencies reported exclusion of several etiologies such as autoimmune hepatitis, but they did not disclose the diagnostic exclusion criteria [3], as is required [56]. Sixth, regulators also interviewed patients, but details of structured questionnaires to circumvent bias were again not provided [3]. Eighth, regulators reported reviewing the medical charts of the patients [3]. This commonly implies that the clinical diagnosis of hepatotoxicity by OEP is approved by the assessing regulators, being correct and well documented.

### 6.7. Liver Histology

Puzzling is the CDC information that 10 patients had liver histology data available at the time of this report, *i.e.*, 11 October 2013, and seven had histology consistent with hepatitis from drug/toxic injury [3]. It seems that the results of the three other liver histologies were not consistent with this type of injury but signified other liver diseases, which were not disclosed. This point of uncertainty remained unanswered in the clinical report [5] but was thoroughly discussed later by our group [9].

### 6.8. Confounders

It is clear that major confounders impede the cases presented by the CDC (Table 8) [3]. Detailed above, these confounders raise the question whether the CDC conclusions were based on valid evidence or rather on speculations. Of note, other regulators have been confronted with confounding variables in HILI cases before, both in the US [65,66,90] and Germany [106,107,108,109,110,111,112,113]. In most of these instances, confounders led to a downgrading to excluded or unlikely causality levels in virtually all examined cases [66,90,107,108,109,110,111,112,113], in the case of kava supported by a German court decision [109]. There is no question that HILI is a major clinical and regulatory challenge [113], a view supported in a recent review article with detailed information of this interesting field and encouraging suggestions for better models of pre-emptive testing of potential hepatotoxic properties of herbs [114].

### 6.9. Variable Case Numbers

Reported case numbers vary substantially. CDC states that on 9 September 2013, the HDOH was notified of seven patients [39]. In their illustrating figure, they present overall 31 patients, namely, 20 patients from May to August 2013, an additional 8 patients in September, and 3 more patients in October 2013 [3]. These figures are at variance with the eight patients reported in the QMC publication of 2014 [5], seven of which are the founding patients [3]. CDC also published that clinicians reported 45 possible cases to the HDOH in response to a public health alert. Of those, 29 have been identified as cases [3]. Of these 29 identified patients, 24 reported using OEP during the 60 days before illness onset. Twelve reported use of OEP and no other DS, and 12 others reported use of OEP and at least one additional DS. Three reported using other DS’s, which were not further specified, but not OEP. Interestingly, information about OEP use was not yet known for two patients, but reasons for such delay remain undisclosed [3].

## 7. Hawaii Department of Health Report

This HDOH report was intended to serve as a final update and to provide findings from the epidemiological investigation and more in depth examination of the events in Hawaii [8]. It is another attempt to clarify the OEP issue that originated at the QMC [5], and it is an update of the preliminary CDC report from 2013 [3]. Details are discussed on 44 patients from Hawaii [8].

### 7.1. Product Identification

Products were identified with differentiation between old OEP and new OEP, according to listed ingredients but without specific causality attribution to the one or the other OEP product [8]. In line with our report (Table 2) [9] are the listed ingredients of the OEP products [8].

### 7.2. Causality Assessment Method

For causality assessment of the cases, the HDOH used the proper version of RUCAM [8], the 2013 version as published by our group [56,113]. On a quantitative basis, HDOH described 10 patients in Hawaii with a probable causality for any OEP [8]. Since a statement to the contrary is not provided, these 10 cases likely included the final 8 QMC cases published by the QMC physicians [5], with resulting invalid causality gradings due to willingly upgrading [6,9]. Therefore, these 8 QMC cases have to be subtracted from the 10 HDOH cases [8], leaving just 2 from the initially published 10 HDOH cases with a probable causality level. Lacking any clinical relevance, HDOH also reported on 24 cases with a possible causality and 2 others with an unlikely causality, but it was not disclosed whether QMC physicians or HDOH epidemiologists made the assessment by RUCAM [8]. All these figures are under conditional acceptance due to multiple confounders (Table 8), also detailed below.

### 7.3. Case Definition

Case definition of the HDOH report [8] was similar to the case criteria established by the CDC [3], again with the same procedural shortcomings as outlined above. For the HDOH cases, work-up for less common causes of acute fulminant hepatitis (*i.e.*, autoimmune markers, HEV serologies) was performed at the discretion of the diagnosing physician, a vague approach [8]. This implies that a standard diagnostic protocol for all cases and all physicians was not available [8].

### 7.4. Inconsistent Data Presentation

HDOH data are presented in a way that is confusing, inconsistent, and contradictory, and implies a high number of probable cases [8]. HDOH epidemiologists reported on 41 “probable” cases out of 44 cases that met case definitions in their first table, whereas their third table presents only 10 cases following causality assessment [8]. This misuse of the term “probable” to refer merely to cases that met their case definition is unacceptable, as it gives a false impression of a high number of “probable” cases by RUCAM causality assessment. It also values high case numbers over data quality.

### 7.5. Comorbidities

Among the reported 44 HDOH cases, the frequency of comorbidity was high [8]. This figure supports the high comorbidity rate published by our group for the QMC patients [6,9] and offsets the statements of the CDC and the QMC that the analyses patients were all healthy before, implying lack of comorbidities [3,5]. With respect to the comorbidity frequencies, it appears that we are dealing with different patient cohorts. In 22 of the 44 HDOH cases (50%), comorbidities were described, with 3–5 comorbidities in 4 patients and 1–2 comorbidities in the remaining 18 patients [8].

### 7.6. Synthetic Drugs as Comedication and Potential Causes

In the HDOH group with 44 patients, comedication by synthetic drugs is a major issue in 31 patients (70.5%), 18 of these used NSAIDs and 13 consumed acetaminophen as Tylenol [8]. This raises the question of how many HDOH cases saw hepatotoxicity due to known hepatotoxic drugs, rather than the assumed liver injury from OEP [8]. The use of these drugs and their potential to cause DILI was not analyzed and published in the CDC report [3] or the QMC report [5]. For many patients, our analyses of the QMC cases revealed substantial comedication by drugs (Table 6) [6,9] and the risk of DILI (Table 1 and Table 3) [6,9].

Despite the striking comedication by drugs in the HDOH patients, the HDOH did not make clear whether each drug received a separate causality assessment by RUCAM to prove or exclude these drugs as causes for the observed liver diseases [8]. Publication of such analyses is mandatory to ensure transparency and balanced conclusions derived from unbiased results [53,58]. Therefore, the overall contribution of drugs in the liver diseases of the HDOH cases remains unresolved.

### 7.7. Additionally Used Dietary Supplements as Potential Causes

Data on the 44 HDOH cases are confusing since eight patients did not use OEP or another defined DS [8]. Most of the remaining 36 cases, namely, 22 patients corresponding to 61.1%, used OEP in combination with another DS. These DS’s were not separately assessed for individual causality [8], despite hepatotoxicity reported for many DS’s [54,55]. In 14/36 cases equating to 38.9%, OEP was used alone but was not stratified regarding specific OEP product or its causality grading [8].

### 7.8. Alcohol Use

Alcohol use as a potential confounder was recorded in 22/44 HDOH (50%) cases, with use of more than four alcoholic beverages per occasion in seven patients [8]. Alcohol consumption was not mentioned in the CDC report [3] or the QMC report [5] and rarely in our analyses of the QMC cases [6,9]. Alcohol is a potential cause for the liver cirrhosis in a patient (case 1) (Table 1) of the assessed QMC cases [9].

### 7.9. Time of Onset

QMC case analyses revealed that patients used the old OEP often for many years prior to the QMC mystery [6,9], an observation also made by the QMC physicians [5]. Yet prior to 22 April 2013 and as illustrated in their second figure, HDOH did not identify a single Hawaiian patient with liver disease due to old OEP [8], confirming nationwide assessments of the US Drug-Induced Liver Injury Network (DILIN) group about liver injury from herbals and dietary supplements [55]. In this DILIN study, as of March 2013, overall 129 patients were enrolled with suspected liver injury after use of various herbs or DS’s, but OEP was not identified in this cohort as a single and specific culprit [8,55], again pointing to the QMC as the primary cause of the OEP dilemma. The 129 DILIN patients each used up to six different DS products and herbs, representing a large patient group with little chance for a single product with a clear hepatotoxic potential to escape. However, the DILIN method used for assessment did not provide individual causality gradings for each patient for each of the products that was consumed [55].

### 7.10. Questionable Illustrative Cases

HDOH presents two cases, presumably their strongest cases, in an attempt to illustrate robust results [8], but this approach failed and provided evidence to the contrary.

#### 7.10.1. Case 1

This patient used an OEP product, though the version of the OEP was not specified, for an unknown period before liver disease emerged with ALT 4120 U/L at her first visit on 6 June 2013 [8]. She reported continued OEP use for another month after the first visit and then stopped, with improvements of symptoms. She restarted OEP use in September and was seen on 17 September with emesis and an ALT 150 U/L, signifying a substantial drop from initial 4120 U/L. She stopped OEP again after hearing media reports in late September and presented to her physician on 9 October with ALT 1494 U/L. A RUCAM score of 8 was attributed to this case, but details are not provided which items were used and what individual scores were provided. Several important questions remain unanswered: (1) Was the period prior to 6 June assessed; or (2) the period 6 June to around 7 July; or (3) periods in September until 17 September; or (4) the period late September to 9 October? For each of these periods, challenge data and resulting scores vary substantially. Variability applies also to dechallenge, where respective scores are also not provided. Additionally, vomiting was reported, which reduces OEP ingestion and influences causality scoring. An even more significant issue is that these conditions appear to have been interpreted as positive reexposure, for which valid evidence is lacking, especially as the version of OEP was not specified and may have been different in the two time periods. Overall, case data as presented are confusing and cast doubt on a probable causality for OEP as claimed by epidemiologists [8]. In such a situation, transparency of case data and RUCAM assessment item by item is mandatory, as postulated before [41].

#### 7.10.2. Case 2

The second case of this report [8] is likely identical with case seven of the QMC publication [5], although data inconsistencies exist between these two reports [5,8], considering also details as extracted from the clinical files of this female patient and summarized by our group (Table 1 and Table 3). For case two, an emergent liver transplantation work-up revealed ductal carcinoma *in situ* of the breast, precluding liver transplantation [8]; these findings were confirmed for the left breast by our reanalysis (Table 1). After having reviewed the clinical records of this patient (Table 1), we agree with the statements of the HDOH report [8] that the patient did not undergo a liver transplantation. This is at variance to the claim of the QMC physicians that their patient (case 7) had received an orthotopic liver transplant [5]. Through this upgrading manoeuvre of severity and transplantation numbers, the QMC physicians attempted to distort the statistics directed to an OEP disaster, in line with their initial biased claims.

According to the HDOH report, their second patient used an unspecified OEP product for an unspecified time and stopped use on 7 September. On 22 September, her ALT was 835 U/L, and a probable causality for OEP was claimed, resulting from a score of 7 [8]. Such a high score also raises questions regarding dechallenge data. The first ALT value was obtained two weeks after OEP use was stopped, which does not allow the evaluation of the natural course of dechallenge, which is assessable only immediately after use of the product is stopped, not weeks thereafter. Lack of information on accurate dechallenge data leads to a score of 0 points [110]. Since causality levels of all eight QMC cases were willingly upgraded by the QMC physicians [9] and distort their published conclusions [5], it is unclear whether the statements of the HDOH report are based on the upgraded causality levels and other incorrect case data. We have serious concerns, however, since the causality assessment yielded a final “probable” score of 7 in both reports [5,8], suggesting that the DHOH used incorrect and biased QMC data without checking these for validity.

Finally, the HDOH epidemiologists appeared to have been unaware that this patient initially claimed to have used Oxy Cleanse. Mentions of OEP did not appear until later, seemingly when the OEP publicity emerged (Table 1 and Table 4). The issue of product identification, purchase proof, and consumption was also debated in court.

### 7.11. Variability of Case Numbers

Case numbers presented in this HDOH report [8] vary substantially from one publication to the other and run the risk of case duplication considering the various reports [3,5,7,9].

### 7.12. Confounders

The HDOH report is confounded by several elements [8]; some of these confounders are presented in a listing compilation (Table 8).

## 8. Food and Drug Administration Report

In its Perspective article of 3 April 2014, the New England Journal of Medicine reported in reference to the QMC liver patients that by February 2014, the FDA had linked 97 liver cases to OEP [4]. This high case number seems to have been an overestimate that exacerbated the stream of misinformation. Presumably, it was overreporting caused by case upgrading and through supplementing some liver cases by all cases with any adverse reaction apart from the liver. At variance to these initial and erroneous 97 FDA cases [4] is the actual FDA research article published in 2015 [7]. This FDA report indeed assumed liver diseases after OEP use in only 55 cases, which were communicated until January 2014 [7]. The erroneously assumed 97 FDA cases will not be further considered in this review article. More important is the actual FDA report, which assumed 55 cases [7]. Among these, the FDA assessed 21 QMC cases from Hawaii and 12 cases from the continental US.

### 8.1. FDA Cases in the Overview

Viewing the earlier 55 cases with liver diseases, the FDA analysis left now 33 liver cases that merit further consideration [7]. In fact, the FDA reviewed 21 medical records provided by the HDOH for the QMC cases and then collected medical records from 12 consumers in the continental United States.

### 8.2. FDA and Its Case Definition

Adverse event reports required evidence of one or more of the following to define an illness: elevated transaminases and/or total bilirubin, jaundice and/or icterus, a diagnosis of hepatitis or acute liver failure, and/or an illness for which the subject underwent liver biopsy and/or liver transplantation [7]. As laboratory results are not quantified and the statements about liver biopsy are vague, this case definition is clearly not in line with mainstream clinical hepatology when liver injury cases by drugs, herbs, and DS’s are to be evaluated [58,100,105,113], substantiating that the FDA is facing challenges during its HILI case assessment [7] similar to those outlined recently [100,114]. The FDA case definition does not account for well-defined hepatotoxicity criteria, such as challenge, dechallenge, comedication, and alternative causes [58,113]. Using the above FDA criteria, any liver injury case assessment likely produced questionable results.

### 8.3. FDA Report and Its Problematic MedWatch System

Little evidence exists that the FDA’s MedWatch system performs well in DILI and HILI cases [65,66]. Major problems include incomplete data sets, insufficient exclusion of alternative causes, missing transparency, and lack of product identification, to name some examples. There is also the note that MedWatch reports have rarely provided sufficient and adequate timely data to permit detection of clusters of serious adverse effects from supplements [4]. The poor quality of the MedWatch reports is easily explained by the qualification of the case reporting individuals, as detailed in the FDA report [7]. Most adverse event reports were submitted by 58 non-health professionals: consumers (53 reports), friends or relatives of consumers (two reports), industry (two reports), others were submitted by a poison control center (one report); and some by 53 health professionals; for three reports, the source remained undetermined.

### 8.4. FDA and the Lack of a Sufficiently Sophisticated Causality Assessment Algorithm

Under the specific heading of causality assessment, the FDA did not make clear whether RUCAM was applied to assess causality for OEP or one of the co-used DS’s in the reported cases [7]. This failure represents a major diagnostic flaw in regulatory causality assessment of suspected HILI or DILI cases. A highly questionable statement is published that FDA concluded that a causal link existed between OEP ingestion and liver injury on the basis of evidence [7]. First, FDA claimed that liver injury reversed after OEP ingestion ceased, but proof was not provided for any of their cases; Second, FDA claimed that other causes of liver disease were systematically excluded, including viral infections, autoimmune disorders, and intoxications—an undocumented claim reiterating previous questionable statements by the CDC, as discussed above; Third, FDA claimed that undocumented results of liver biopsies suggested DILI, a point of uncertainty discussed also for the CDC report above. For all this claimed evidence, the FDA failed to provide support in the form of complete and transparent case data [7]. 

### 8.5. FDA and the 21 Honolulu QMC Liver Cases

The FDA claimed to have reviewed 21 medical records of patients with liver disease at the QMC in Hawaii, who had ingested OEP together with at least one DS, but failed to state that the individual DS was not submitted to a separate causality assessment to consider the concomitant use of various DS’s [7]. It appears that the FDA did not recognize in the QMC files, which they had obviously reviewed [7], that the QMC patient causality scores had been unjustifiably upgraded, many alternative causes, various unrecognized but treatable infections by hepatitis viruses, and biased conclusions [6,9]. Therefore, statements of the FDA regarding the QMC cases do not provide a reliable basis for causality.

### 8.6. FDA and the 12 Nationwide Liver Cases

The FDA collected medical records from 12 consumers in the continental US, who reported liver disease after ingesting OEP [7]. No details of these cases and no results of a RUCAM causality assessment were provided, preventing analysis as to whether these cases are valid liver cases by OEP.

These 12 case patients resided in nine states: California (three reports), Ohio (two reports), and with one single report in each Arizona, Kentucky, New York, Minnesota, Rhode Island, Virginia, and West Virginia [7]. These figures illustrate that only Hawaii [3,5,6,7,8,9] and no other US state had a problem of case accumulation [7], only the QMC [3,5,6,9] and no other major medical center or transplant center [7]. There is no question that the OEP liver problem is located solely at the QMC. Prior to 2013, nationwide analyses of 129 patients with liver disease by herbal dietary supplements done by the DILIN group specified no problem with OEP in these patients who took a total of 217 products, maximum 6 products per consumer [55]. Additionally, analyzing adverse event reports of OEP with liver disease submitted to FDA showed no or only single cases as “background noise” in the years 2010 to 2012, with a dramatic increase of cases in 2013 [7], due to the surge in publicity around OEP in 2013 and triggered by incomplete case data at the QMC in Honolulu [6,9].

### 8.7. Variability of Case Numbers

This report [7] adds to the cases of the other reports [3,4,5,6,8,9] and increases the case number variability and the risk of case duplication.

### 8.8. Confounders

Confounding variables prevail in this report [7] and are partially presented in a listing compilation (Table 8).

## 9. Limitation of this Review

The present analysis has its limitations since various regulatory reports are reviewed that are based on QMC physicians’ case evaluations, which did not meet accepted standards and appear not to have considered many alternative causes, and also included unjustifiable increased causality assessments. There is a strong likelihood that these circumstances biased the regulatory case assessments and led to invalid conclusions.

## 10. Conclusions

The mysterious accumulation of liver cases at the Queen’s Medical Center in Hawaii in the summer of 2013 turned out to be a localized problem at this Medical Center and not a general problem of a dietary supplement such as OEP. Indeed, with their own mishandling of case data and causality upgradings, the QMC physicians undermined their initial claim that the liver disease of their patients at the QMC is caused by OEP. Patients suffered also from various alternative diseases, which could have been treated but remained untreated because they were not diagnosed captured. These flaws created by the QMC were not recognized during regulatory case reviews and became public only recently. This delay caused invalid regulatory case assessments and public statements that were based on these unacceptable clinical evaluations and conclusions by the QMC and not on actual data providing results and conclusions to the contrary. These conditions call for an official hearing to investigate the overt problems, to provide clarity, and to avoid similar problems in the future.

## Figures and Tables

**Table 1:** Established and final diagnoses of the Queen’s Medical Center (QMC) patients (cases 1–8). Causality for all products was assessed by us using the Roussel Uclaf Causality Assessment Method (RUCAM) as described in earlier reports, which present many additional details and how case data were provided [6,9].

Patients	Final Diagnoses and Alternative or other Important Diagnoses	References
Case 1	**Final diagnosis**	[9]
	Decompensated liver cirrhosis by alcohol, AAP, or HEV; highly probable hepatitis E.	
	Decompensated liver cirrhosis of clinically unassessed etiology, preexisting and likely due to alcohol, acetaminophen, or HEV. HEV highly probable, suggested by high ALT values, lack of ALT dechallenge, and patient’s specific HEV risks. Presumably, physicians missed the tentative HEV diagnosis and effective antiviral drug therapy by ribavirin prior to OLT.	
	**Alternative diagnoses**	
	1. Suspected acetaminophen hepatotoxicity, connected with alcohol use.	
	Acetaminophen hepatotoxicity and liver cirrhosis with alcohol as predisposing factor remained unconsidered by regulatory or clinical assessments, as was the possible specific treatment by *N*-acetylcysteine to circumvent ALF and OLT.	
	2. Possible acute acalculous cholecystitis.	
	3. Possible resolving acute hepatitis by CMV and VZV coinfection.	
	4. Morbid obesity.	
Case 2	**Final diagnosis**	[9]
	Acute liver failure by accidental chronic acetaminophen overdose.	
	This diagnosis was clinically missed, and timely specific therapy by *N*-acetylcysteine was not given which could have prevented OLT. In his liver histology report, the pathologist reminded the physicians that consideration of AAP toxicity is warranted, a suggestion the physicians did not seriously consider. Mainstream opinion suggests this kind of treatment in any case of acute liver failure, independent of the cause. Tentative probable causality for AAP.	
	**Alternative diagnoses**	
	1. Acute liver failure by the known potentially hepatotoxic nonsteroidal antiinflammatory drug ibuprofen.	
	Tentative possible causality for ibuprofen, a known hepatotoxic NSAID. Some key features are poorly documented.	
	2. Possible resolving acute EBV infection.	
	Documented are high anti-EBV IgG titers, suggesting a resolving acute EBV infection in which IgM has vanished. Titers of anti-EBV IgG were not followed in the further course to confirm or disprove the assumed resolving acute EBV infection.	
	3. Possible, not sufficiently excluded hepatitis E.	
	High ALT values and clinical features are compatible with and suggestive of HEV infection. HEV exclusion is fragmentary and likely done using antibody tests that are not FDA approved due to overt problems of specificity and sensitivity. HEV PCR was not assessed in blood, stool, and the explanted liver.	
	4. Morbid obesity.	
Case 3	**Final diagnosis**	[9]
	Acute hepatitis by HBV and VZV coinfection.	
	The medical records substantiate the diagnosis of acute HBV and VZV coinfection: anti-HBs and anti HBc were positive; HBV immunization is not documented. Acute VZV hepatitis is ascertained by positive tests for anti-VZV IgM and IgG; microgranulomata are described in virus hepatitis including VZV. Both infections might have been occurred around or after June 2013. In June, the patient was on a trip in California for one week. Both diagnoses were missed, also potential therapy; prolonged clinical course under lacking therapy. Recovery was not documented in the files [9] and not published [5].	
	**Alternative diagnoses**	
	1. Acute cholecystitis.	
	Ultrasound and abdomen MRT presented results of a thickening of the gallbladder wall, according to assessors suggestive of cholecystitis with pericholecystic fluids and consistent with acute *versus* chronic gallbladder disease. Clinical signs including prolonged abdominal pains and prolonged increase of LTs are compatible with cholecystitis. No therapy is documented in the files.	
	2. Possible DILI by acetaminophen.	
	3. Obesity.	
Case 4	**Final diagnosis**	[9]
	Acute HSV hepatitis due to genital herpes; acute cholecystitis with multiple gallbladder stones.	
	Clinical symptoms, high ALT values, liver histology, and antiviral HSV treatment by prolonged high dosed aciclovir therapy for genital herpes are suggestive of acute HSV hepatitis.	
	**Alternative diagnoses**	
	1. Resolving acute CMV hepatitis.	
	Anti-CMV IgG titers increased with normal IgM titers, a constellation compatible with a resolving acute CMV infection where IgM had already disappeared. It was forgotten to determine IgG in the further clinical course with assessment of quantitative titers to evaluate titer changes.	
	2. Acute hepatitis, preferentially by hepatitis E virus (HEV) infection (not yet excluded).	
	Liver histology suggests acute virus hepatitis and testing for HEV, but clinicians discounted this advice.	
	3. Overweight.	
Case 5	**Final diagnosis**	-
	Unlikely OEP hepatotoxicity. Possible hepatitis by HEV, EBV, HSV, or VZV, none yet excluded.	
	MedWatch report did not specify that one of these infections were ruled out. Limited data from MedWatch report, not reported by a physician but by a pharmacist. Raw clinical and case data were not provided by the QMC.	
	**Alternative diagnoses**	
	1. Fatty liver.	
	Diagnosis based on CT report.	
	2. Unspecified pain syndrome.	
	Question of additional, yet undeclared pain medication.	
	3. Obesity.	
Case 6	**Final diagnosis**	-
	Undetermined due to lack of raw data.	
	Raw clinical and case data were not provided by the QMC.	
	**Alternative diagnoses**	
	1. High alcohol consumption.	
	Alcohol use: up to 10–12 beers per occasion.	
	2. HEV not excluded.	
	Anti-HEV IgM, anti-HEV IgG, and HEV PCR not assessed.	
	3. Obesity.	
Case 7	**Final diagnosis**	-
	Undetermined due to uncertainty of used product.	
	Uncertainty about the used DS does not allow a causal attribution to any DS (Table 4): Oxy Cleanse (OC) or OxyELITE Pro (OEP). The probable causality initially claimed for OEP by the QMC physicians [5] cannot be accepted as valid any more due to unjustifiable upgrading of the causality levels of all eight QMC patients including this case 7, as admitted at a trial [9]. As an exclusion diagnosis, a thorough exclusion of other potential causes was not done and not documented in the analyzed medical records.	
	**Alternative diagnoses**	
	1. Suspected hepatitis by varicella zoster virus (VZV) infection.	
	Anti-VZV IgG was positive, but titer changes in the further course were not assessed. VZV PCR was not assessed in the blood and in the liver at autopsy.	
	2. Suspected hepatitis E virus (HEV) infection.	
	HEV remained unconsidered by the QMC physicians. HEV PCR was not done in blood, stool, and the liver at autopsy. Anti-HEV IgM and IgG antibodies were not determined, not with FDA unapproved or approved HEV antibody tests.	
	3. Suspected Wilson disease (WD).	
	WD is a known cause of ALF, which was not carefully excluded by QMC physicians.	
	4. Obesity.	
Case 8	**Final diagnosis**	[6]
	Recurrent toxic hepatitis by naproxen (Aleve) overdose 8–9/2013 and 4/2014, with lacking causality for dietary supplements including both OEP products.	
	**Alternative diagnoses**	
	1. Acute acalculous cholecystitis associated with gallbladder sludge.	
	Suggested by clinical symptoms and imaging data.	
	2. Acute hepatitis, preferentially suspected hepatitis E virus infection (not yet excluded).	
	HEV not considered as potential cause.	
	3. Nonalcoholic fatty liver disease (NAFLD) due to morbid obesity with BMI 40–45 kg/m^2^.	
	4. Suspected DILI by one of the abundant used synthetic drugs.	
	5. Well documented multimorbidity.	
	6. Chronic pain syndrome, headaches, and intractable migraine.	

AAP: acetaminophen; ALF: acute liver failure; ALT: alanine aminotransferase; BMI: body mass index; CMV: cytomegalovirus; CT: computed tomography; DILI: drug induced liver injury; DS: dietary supplement; EBV: Epstein Barr virus; HBc: hepatitis B core; HBs: hepatitis B surface; HBV: hepatitis B virus; HEV: hepatitis E virus; FDA: Food and Drug Administration; HSV: herpes simplex virus; LTs: liver tests; NSAID: non-steroidal antiinflammatory drug; MRT: Magnetic Resonance Tomography; OC: Oxy Cleanse; OEP: OxyELITE Pro; OLT: orthotopic liver transplantation; PCR: polymerase chain reaction; QMC: Queen’s Medical Center; WD: Wilson disease; VZV: varicella zoster virus.

**Table 2:** Formulas of OEP products used by the liver patients at the QMC.

Product	Main Ingredients *	Other Ingredients *	Total Weight
Old OEP: OxyELITE Pro	Caffeine (100 mg), Bauhinia purpurea L. Extract, Bacopa Monnieri Extract, 1,3-Dimethylamylamine HCl (20 mg), Cirsium Oligophyllum Extract, Yohimbe Extract	Modified Starch, Gelatin, Vegetable Stearate, Silicon Dioxide, Red 3, Blue 1, Red 40, Titanium Dioxide Color	1 capsule: 380 mg
New OEP (I): OxyELITE Pro New Formula	Caffeine (135 mg), Bauhinia purpurea L. Extract, Aegeline (40 mg), Norcoclaurine HCl, Hemerocallis fulva Extract, Yohimbe Extract	Modified Starch, Gelatine, Vegetable Stearate, Silicon Dioxide, Red 3, Blue 1, Red 40, Titanium Dioxide Color	1 capsule: 405 mg
New OEP (II): OxyELITE Pro Advanced	Caffeine (100 mg), Cynanchum auriculatum Extract, Olea Europaea Extract, Aegeline (50 mg), Yohimbe Extract, Coleus Forskohlii Extract	Gelatine, Modified Starch, Silicon Dioxide, Vegetable Magnesium Stearate, Red 3, Blue 1, Red 40, Titanium Dioxide Color	1 capsule: 460 mg
New OEP (III): OxyELITE Pro Super Thermo	Choline Bitartrate, l-Carnitine-Tartrate, Caffeine (125 mg), Aegeline (90 mg), Norcoclaurine HCl, Yohimbe Extract, Oleoylethanolamide, Eriobotrya Japonica Extract	Malic Acid, Silicon Dioxide, Sucralose, Acesulfame Potassium, natural and artificial Flavors	1 Scoop: 2167 mg

*: Ingredients are listed in descending order of predominance by weight; data are as provided by the manufacturer, from a previous report [9].

**Table 3:** Narratives of the QMC patients (cases 1–8). Information of sex and age of each patient was derived from the QMC report [5], because the files analyzed by our group were all redacted with details published recently [6,9]. Causality for all products was assessed by us using RUCAM.

Patients	Narratives	References for Additional Details
Case 1 Male, 22 years	**Narrative**	[9]
	Patient with liver cirrhosis and ascites of clinically unassessed etiology but possibly related to alcohol or acetaminophen use, or HEV. Significant outdoor activities with HEV risk: hunter, wild hog meat consumer, and coffee farmer. History of illicit drug and heavy alcohol use/abuse some years ago, with present monthly use of 6–12 beers or a 6 pack of beer and 1–2 shots of hard liquor each time the patient drank. First symptoms of illness emerged within 24 h after a restaurant meal. At hospital admission on 25 August 2013: ALT 1970 U/L, AST 1308 U/L, ALP 107 U/L, and bilirubin 33.6 mg/dL. Significant PMH (Table 5). Documented prior use of synthetic drugs (Table 6). Additionally, acetaminophen-oxycodone in early 2013 for pain in neck and paresthesia in the arm, possibly an early extrahepatic, neurological manifestation of HEV myelitis with polyradiculopathy, bilateral brachial neuritis, or peripheral neuropathy, which can overshadow the liver injury. Use of overall 14 DS’s (Table 5): old OEP use for one year, intermittently on an off and on basis and stopped 3 weeks prior to admission; subsequently, new OEP, Amplified Wheybolic Extreme-60, and Versa-1, all used for only one week until symptoms emerged two weeks prior to admission, unlikely causes of decompensated liver cirrhosis and ALF due to short duration of use.	
	Clinical features and laboratory results are highly suggestive of HEV infection, but lacking results of HEV PCR and anti-HEV IgG; invalid negative anti-HEV IgM as assessed by a test that is not FDA approved. Negative anti-CMV IgM and anti-VZV IgM. Positive anti-CMV IgG and anti-VZV IgG without assessed IgG titer changes in further course; possibly resolving acute hepatitis by CMV and VZV coinfection. ALT decrease not continuously straight but variable and undulating with intermittent spikes, possibly caused by limited HEV episodes or intermittent acute cholecystitis bouts. Positive Murphy sign, imaging data with up to 1 cm thickening of the gallbladder wall and surrounding fluids, suggestive of acute acalculous cholecystitis with discussed surgical consultation. Patient required OLT: decompensated liver cirrhosis with ascites, confirming prior CT result. Explanted liver not assessed for HEV PCR. After OLT, increased LTs by rejection or HEV episodes.	
	Causality assessment: Poor case data quality. Used algorithm applicable only to acute and not chronic liver injury such as decompensated liver cirrhosis. Tentative excluded causality for old and new OEP, all additional DS’s, and AAP. Intermittent use of old OEP and the large interval of 2–3 weeks from stop to admission impedes a valid assessment of the natural dechallenge course of ALT. Diagnoses: Decompensated liver cirrhosis by alcohol, AAP, or HEV; highly probable hepatitis E.	
Case 2 Female, 31 years	**Narrative**	[9]
	Patient with initial admission on 25 August 2013 and later transferal to the liver transplantation center due to ALF with liver transplantation on 9 September 2013. The day prior to admission: ALT 1416 U/L, AST 936 U/L, ALP 107 U/L, bilirubin 7.9 mg/dL. Two week history of vomiting and inability to eat prior to admission, yellowing of her eyes. Significant PMH (Table 6). Use of multivitamins, multiple DS’s including possibly OEP (Table 5), also multiple synthetic drugs and phentermine (Table 6).	
	High titers for anti-EBV IgG, suggestive of resolving acute EBV infection with already normal IgM; anti-EBV IgG titers not assessed in the further course to confirm the resolving EBV infection. Anti-HEV IgM and IgG negative, but applied antibody tests not described with their characteristics of sensitivity and specificity. HEV PCR not done in blood, stool, and liver. Upon ultrasound examination, initially some intrahepatic biliary duct dilatation in the right hepatic lobe and about the gallbladder fossa, but not persisting. Liver histology: Massive confluent necrosis, the etiology of the acute liver failure is uncertain, but consideration of acetaminophen (AAP) toxicity is warranted given the patient’s history of gastric sleeve/gastric bypass procedure—a known risk factor for such toxicity.	
	Causality assessment: Insufficient data quality. Excluded causality for new OEP, phentermine, multivitamins, and various DS’s, probable for acetaminophen, possible for ibuprofen, and likely for the multiple unidentified headache medicines. Diagnosis: Acute liver failure by accidental chronic overdosed acetaminophen.	
Case 3 Female, 43 years	**Narrative**	[9]
	The patient used several drugs (Table 6), including ferrous sulfate in 6–7/2013: prescription is well documented, but not the indication, likely blood-loss-related anemia of unknown etiology. On 12 June 2013, start with 50 doses of tramadol-acetaminophen with unknown daily dose and duration, likely for pain relief, which may have emerged as initial symptoms of the later established acute hepatitis by HBV and VZV coinfection. Normal blood tests in 7/2013 claimed, but details not documented. On 12 August 2013, admission with diagnosis of obstructive jaundice, anemia, and abnormal LTs. Subsequent diagnoses included acute liver failure (lacking criteria), hepatitis, or simple transaminitis. OEP use was mentioned only late in the clinical course. Inconsistently described use of new OEP for 3 or 6 weeks, or a couple of months, possible prior use of old OEP for 3 and 2 years; proof of purchase not documented, also no documentation in the PCP files. On admission, AST 736 U/L, ALT 636 U/L, ratio AST/ALT constantly >1, ALP 156 U/L, and bilirubin 6.5. Hgb 9.9 g/dL, cholesterol 272 mg/dL, BMI 33 kg/m^2^. Without specific treatment, her liver values failed to decline, as ALT was with 867 U/L on 30 November 2013 even higher than at admission, suggesting ongoing hepatitis. Unclear outcome in face of missed diagnosis and lacking specific therapy.	
	Imaging report suggests acute *versus* chronic gallbladder disease. Immunization records: empty. Hepatitis B serologies obtained after admission and later establish acute hepatitis B virus infection: anti-HBs were twice positive within 4 weeks, while anti-HBc undulated and was positive, negative, and finally negative/positive. Hepatitis B surface antigen was already negative. Coinfection with acute VZV infection, confirmed by positive anti-VZV IgM and IgG. Anti-HEV IgM negative by a kit from Focus Diagnostics that has not been approved by the FDA. HEV PCR assessment was not done in blood or stool. Liver histology includes microgranulomata and shows acute confluent necrosis.	
	Causality assessment: Excluded causality for old and new OEP, unlikely for ferrous sulfate, possible for AAP, and excluded for additional drugs. Diagnosis: Acute hepatitis by HBV and VZV coinfection.	
Case 4 Female, 54 years	**Narrative**	[9]
	Symptoms started with pruritus on 18 May 2013 and progressed to jaundice, dark urine, nausea, stomach pain, and rash, before LTs were assessed. On 6 December 2013: ALT 1750 U/L, AST 1847 U/L, ALP 190 U/L, bilirubin 14.1 mg/dL. ALT declined slowly, undulating and with spikes, suggesting some hepatitis or cholecystitis episodes. Documented multimorbidity (Table 5) and multimedication (Table 6). Documented use of OEP and abundant herbs and DS’s. After the liver illness, this patient continued to purchase dietary supplements, including new OEP in 9/2013 as well as Hydroxycut, which might have been used also before. Prolonged and high dosed aciclovir therapy, genital herpes as likely origin of HSV hepatitis.	
	Abdominal ultrasound with contracted gallbladder and wall thickness of 8 to 9 mm, multiple stones, or sludge. Negative Murphy sign. MRCP: spleen 10.8 cm, trace periscystic fluid and ascites. Autoimmune parameters negative. Negative results for anti-HAV IgM, anti-HAV total, Monoscreen EBV antibody test, HBsAg, anti-HBc IgM, and anti HCV; HBV and HCV PCR not done. Anti-CMV IgM negative, anti-CMV IgG positive but titer changes in the further course not assessed. HEV, HSV, and VZV not assessed. Liver histology report mentions severe cholestasis and that the most likely etiologies include adverse drug reaction, acute viral hepatitis, and AIH, testing for HEV can be considered.	
	Causality assessment: Excluded causality for the old and new OEP and many additional DS’s, unlikely for drugs including AAP. Diagnosis: Acute HSV hepatitis due to genital herpes; acute cholecystitis with multiple gallbladder stones.	
Case 5 Female, 34 years	**Narrative**	-
	Patient with two weeks nausea, decreased appetite, mild epigastric pain, brown urine and jaundice. On 2 June 2013, ALT 1244 U/L. Consumed an unspecified OEP product with an unknown dose for 3 months. Used product was not available for evaluation. OEP use in combination with linoleic acid and General Nutrition Corporation (GNC) protein shakes. Prolonged clinical course [5], suggestive of an infection by a not yet excluded hepatitis virus. Report from a pharmacist with vague and limited information. Pain medication (Table 6).	
	Causality assessment: For OEP: challenge + 2 points, for dechallenge due to lack of information and long time interval between stop of use and first ALT value 0 points, for DS comedication −1, for insufficient exclusion of alternative causes +1, total +2 points corresponding to an unlikely causality, not considering possible alternative causes of virus infections by HEV, EBV, HSV, or VZV, none yet excluded (Table 1). Diagnosis: Unlikely OEP hepatotoxicity. Possible hepatitis by HEV, EBV, HSV, or VZV, none yet excluded.	
Case 6 Female, 35 years	**Narrative**	-
	No raw data available, limited access to other data. Alcohol use: up to 10–12 beers per occasion.	
	Causality assessment: Not done. Diagnosis: Undetermined due to lack of raw data.	
Case 7 Female, 48 years	**Narrative**	-
	This patient reported prior use of Oxy Cleanse (OC) as documented in the medical charts at hospital admission (Table 4). Significant PMH (Table 5) with prior use of various synthetic drugs (Table 6). Upon our reassessment of her case files, we found that she had a localized cancer of the left breast. This diagnosis remained undisclosed in the QMC case report [5]. Our analyses revealed that OLT was refuted due to her malignancy and in fact was not performed, well documented in the medical records of the QMC. This was not attended to by the QMC physicians, who stated in their case report that the patient did receive an OLT [5].	
	The patient initially received NAC which could have altered the natural ALT course after product cessation and impeded assessment of dechallenge. She was diagnosed with ALF, which she survived for 17 days [5]. Hospital admission with prior symptoms for about 3 weeks: nausea, vomiting, diarrhea and flu-like symptoms, so gastroenteritis was initially suspected. The patient has been on OEP for 3–6 weeks according to variable statements in the files, but the exact date of beginning OEP remained undisclosed. During OEP use, she experienced a dramatic weight loss of 1 pound daily, which persisted when she terminated OEP use on 7 September 2013, possibly caused by preexisting or developing liver disease. On 22 September 2013, ALT 986 U/L. Dechallenge ALT features as key diagnostic items were not assessable since first laboratory values were obtained only on 22 September 2013, two weeks after cessation. Despite OC or OEP cessation, synthetic hepatic dysfunction deteriorated in the further clinical course associated with a lack of clinical improvement. These conditions may indicate that the ALF was not due to OC or OEP but causally related otherwise. As an example, WD and HEV were not excluded, and claims that acute VZV was excluded were not substantiated by negative VZV IgM in face of positive IgG. Good alternative diagnoses may include WD, HEV, and acute VZV; for all these three diseases effective therapies exist.	
	Causality assessment: Regarding OC or OEP use, inconsistencies are documented in the files. First, it is unclear whether OC was consumed rather than OEP. For OEP, there is no documented proof of product purchase in a regular shop *versus* via internet. After her death, claims focused on two bottles of OEP that were found, one was empty and the other one was sealed. However, she might not have lived alone in her apartment/house, and it is not clear whether these two bottles were those of the patient. Documented in the files is also that she was not the only one in the family who used OEP. There is also the documentation that she distributed OEP to other family members, none of these were reported as becoming sick. Therefore, retrospect assessment of her OC or OEP use is difficult and disputed. Diagnosis: Undetermined due to uncertainty of used product.	
Case 8 Female, 34 years	**Narrative**	[6]
	Increased aminotransferases since 5 September 2013 of initially undetermined etiology. Her significant PMH (Table 5) included chronic multipain syndrome with headaches and intractable migraine is also well documented in the files, with headaches originating in her childhood. Consequently, an abundancy of pain medications is documented which included the hepatotoxic NSAID naproxen and previous multimedication (Table 6). As a typical NSAID, naproxen is known for its potential hepatotoxicity. The usual dose is 250–500 mg taken orally twice a day. As recommended by her physician, the patient took naproxen at daily overdoses of at least 1500 mg, and this treatment now was identified as culprit of her liver disease. Actually, five months after the first disease onset, overdosed naproxen was reused to treat daily headaches for a month, and this treatment again caused scleral icterus as at the first disease bout, confirming this drug as the causative agent.	
	Assessing conditions are further complicated by the use of multiple DS‘s including OEP (Table 4). The patient stopped the use of new OEP, because she was feeling unwell, possibly at around 3 September 2013 when she first noted dark urine and experienced symptoms of fatigue, nausea, abdominal pain, and anorexia, followed by jaundice of her skin and eyes on 7 September 2013. At first presentation to her PCP on 5 September 2013, her LTs were increased, with ALT 633 U/L, AST 329 U/L, ALP 123 U/L, and bilirubin 1.4 mg/dL. In the further course of the disease, ALT values were variable in a range between 105 U/L and 794 U/L, which represented a second peak, and normalized within 84 days on 1 December 2014. The variable ALT values are suggestive of an ongoing liver disease; of hepatitis E, which was forgotten to be excluded; of a not yet resolved acute acalculous cholecystitis with gallbladder sludge, which was suspected on clinical grounds, the positive Murphy sign, and the ultrasonic description of an abnormal appearance of the thickened gallbladder wall; of the action of still used synthetic drugs. Reuse of the new OEP was reported on 19 September 2013, but concurrent use of potentially hepatotoxic synthetic medications, with their documented discontinuation only on 30 September 2013, impedes a valid assessment; in particular, the claimed positive reexposure test result for OEP is not substantiated due to these confounding variables.	
	Causality assessment: Unlikely and excluded causality for new and old OEP, excluded for the other DS’s. Lack of appropriate drug use details impedes a valid assessment of causality for most drugs. Diagnosis: Recurrent toxic hepatitis by naproxen overdose.	

ALF: acute liver failure; AAP: acetaminophen; ALP: alkaline phosphatase; ALT: alanine aminotransferase; AST: aspartate aminotransferase; BMI: body mass index; CMV: cytomegalovirus; DILI: drug induced liver injury; DS: dietary supplement; EBV: Epstein Barr virus; HAV: hepatitis A virus; HBc: hepatitis B core; HBs: hepatitis B surface; HBV: hepatitis B virus; HCV: hepatitis C virus; HEV: hepatitis E virus; HSV: herpes simplex virus; LTs: liver tests; NAC: N-acetylcysteine; NASH: non-alcoholic steatohepatitis; NSAID: non-steroidal antiinflammatory drug; OC: Oxy Cleanse; OEP: OxyELITE Pro; OLT: orthotopic liver transplantation; PCP: primary care provider; PCR: polymerase chain reaction; PMH: past medical history; RUCAM: Roussel Uclaf Causality Assessment Method; VZV: varicella zoster virus.

**Table 4:** Dietary supplement use of the QMC patients (cases 1–8).

Patients	Documented or Assumed Use of Dietary Supplements	References
Case 1	Used overall 14 DS’s, with some information for 4 DS’s: old OEP use for one year, intermittently on and off basis, and stopped 3 weeks prior to admission; subsequently, new OEP, Amplified Wheybolic Extreme-60, and Versa-1 were used for only one week until symptoms emerged two weeks prior to admission. According to purchase records and additional medical records, he consumed since March 2013 prior to liver illness these 10 DS’s: Pro Performance AMP Wheybolic Extreme 60, CLK with Conjugated Linoleic Acid (CLA)/ Super Hd Combo Kit; Ripped Freak; Kre-Alkalyn EFX; Pump-HD; Green Coffee Bean; Super HD; CLK; and Beyond Raw Chocolate Re-Grow.	[9]
Case 2	Documented that the patient mentioned multivitamins prescribed by her PCP, but denied despite multiple specific questionings use of any DS at numerous occasions prior to OLT. There is also a note that she might have used new OEP for 3 or 6 weeks or a couple of months, possibly also since beginning of July 2013 and until two weeks before admission (vomiting, inability to eat) and phentermine for weight loss. Consumers purchase documents for OEP were not provided. The purchase records and medical records only support the consumption of the following DS’s prior to liver illness: Mega Men Perform & Vitality; Mega Men Sport Vitapak; Amino Energy; Stby Isopure; and Alph Isopure. No customer purchase records for OEP available. The information on this case as documented in the clinical records is inconsistent, fragmentary, and difficult to assess since it appears that the patient did not tell correct details; results of this assessment are therefore tentative.	[9]
Case 3	OEP use was mentioned only late in the clinical course. Inconsistently described use of new OEP for 3 or 6 weeks, or a couple of months, possible prior use of old OEP for 3 and 2 years; proof of purchase not documented, also no documentation in the PCP files.	[9]
Case 4	Regarding documented use of herbs and DS’s, she has been on numerous undefined herbal supplements for months. This patient’s medical history states that the patient was taking the DS Amberen, *Garcinia cambogia*, Super HD, Raspberry Ketone Chews, Vita Chews, and HCA Supreme. The medical records also noted that the patient may have taken some other DS’s, but she does not know the names of all of these. At the time of illness, the patient reported being on multiple undefined herbal supplements for months. The purchase history of DS’s before the liver illness includes not only OEP but also Vitamin Code Raw Calcium, Mega V products energy products, Hydro Pure, Amp 100% Whey Protein, Pre-Diet Cleanse, Meta Ignite, R3 Extreme Chrome, C4 Xtreme Blue, CLK, Compound 20, and many others. After the liver illness, this patient continued to purchase dietary supplements, including new OEP in 9/2013 as well as Hydroxycut, which she might have used also before, Total Body Rapid Cleanse Renew, Keto-Xt, and Premium Detox 7 day Cleanser.	[9]
Case 5	Used unspecified OEP product for 3 months in combination with conjugated linoleic acid and GNC protein shakes.	-
Case 6	No raw data available. Used Alli (Orlistat).	-
Case 7	Medical records of the QMC documented in the beginning of the hospital stay that the patient reported the use of Oxy Cleanse (OC). Following discussion of OEP in the media and along with the upcoming OEP discussions in the media and QMC physicians with their patients, the DS used switched from Oxy Cleanse to the similar word OxyELITE Pro in the medical records. Purchase documents were not provided by the patient and her family members. At court, this uncertainty remained. This was not outlined in the QMC publication, which claimed OEP use and assessed causality for this DS and not for OC [5].	-
Case 8	Documentation of DS use prior and subsequent to disease onset is inconsistent but includes four different DS’s at different times and in different combinations. Among these are GNC bleaching protein powder and GNC woman’s active multivitamin, which were taken each for at least 2.5 years without any side effects, but the date of cessation is not documented. In addition, the well tolerated use of the first OEP product, the old OEP, is documented for approximately 2.5 or 3 years with reported cessation sometime in 7/2013 and switching to a second OEP formula, the new OEP. The patient stopped the use of the new OEP, because she was feeling unwell, possibly at around 3 September 2013 when she first noted dark urine and experienced symptoms of fatigue, nausea, abdominal pain, and anorexia, followed by jaundice of her skin and eyes.	[6]

DS: dietary supplements; OC: Oxy Cleanse; OEP: OxyELITE Pro; OLT: orthotopic liver transplantation; PCP: primary care provider; QMC: Queen’s Medical Center.

**Table 5:** Past medical history of the QMC patients (cases 1–8).

Patients	Documented Prior and Present Diseases	References
Case 1	Asthma, suspected prior alcohol abuse, suspected cervical spinal syndrome or neurological HEV prodromi, atopia, fungal infections on both feet. Morbid obesity, BMI maximum 42.9 kg/m^2^.	[9]
Case 2	Bariatric surgery for morbid obesity, fatty liver, asthma (as child), hypertension, kidney stones left side, ovarian cyst right, cholecystolithiasis, laparoscopic cholecystectomy, choledocholithiasis-sphincterotomy, migraine, fracture right ring finger, treatment with ibuprofen, disc bulges. Morbid obesity, BMI 47 kg/m^2^.	[9]
Case 3	Unspecified pain syndrome, treated by AAP. At admission: anemia. Obesity, BMI 33 kg/m^2^.	[9]
Case 4	Morbidities include recurrent HSV infections, gallbladder stones, essential hypertension, menopause, hypercholesterolemia, insomnia, mild cataracts, polyp in rectum, pseudomelanosis coli, anxiety, migraine, cancer of left breast, with local simple mastectomy. Overweight, 25.6 kg/m^2^.	[9]
Case 5	Asthma. Unspecified pain syndrome. Obesity.	-
Case 6	No raw data available. Alcohol abuse with 10–12 beers at an occasion. Obesity.	-
Case 7	Remote methamphetamine use and alcohol abuse. Documented are earlier multiple hair and urine tests. Obesity. For the past decades, multiple injuries and illnesses are documented in the files, including multiple staphylococcus abscesses, E. coli in the blood, disc disease at L4–5 and L5–S1, left shoulder impingement syndrome, acromioplasty. Documented as Problem List includes: Nail avulsion of finger, laceration, open FX finger, train of lumbar region, Tinea pedis, Acne, strain of the knee, lump breast, current tear knee, medial meniscus, arthralgia of knee, contusion of the chest, and abrasion of back. Obesity, BMI 31.7 kg/m^2^.	-
Case 8	Morbid obesity, nonalcoholic fatty liver disease (NAFLD); attention deficit hyperactivity disorder (ADHD); schizophrenia; chronic pain syndrome, headaches, migraine (intractable); phobia; epilepsy and recurrent seizures; asthma; chronic otitis externa right ear; obstructive sleep apnea syndrome; latent tuberculosis; multiple tattoos; kidney stones; menorrhagia; esophageal reflux; lumbar disc degeneration; surgical history of insert posterior spine process distract device, spinal cord stimulator, laminectomy decompressive up to two lumbar segments; two surgeries L3–4, L4–5, L5–L1; partial meniscectomy left knee; and others. Morbid obesity, BMI 40–45 kg/m^2^.	[6]

AAP: acetaminophen; ADHD: attention deficit hyperactivity disorder; DS: dietary supplement; NAFLD: Non-alcoholic fatty liver disease.

**Table 6:** Prior and current medication by synthetic drugs of the QMC patients (cases 1–8).

Patients	Documented Past and Current Medication	References
Case 1	Inhalation and synthetic medications for asthma. Vicodin (acetaminophen-hydrocodone), Percocet (acetaminophen-oxycodone); Bactrim (Trimethoprim and Sulfamethoxazole).	[9]
Case 2	Tylenol (acetaminophen) contained in an empty bottle found in her car, large amounts of ibuprofen, possibly other unidentified drugs or DS’s, lacking any further details. “Lot of unidentified medications for headaches”.	[9]
Case 3	Before onset: acetaminophen-tramadol, 50 doses; ferrous sulfate. After onset: Amoxicillin, ibuprofen, clarithromycin, omeprazole, and promethazine.	[9]
Case 4	Aciclovir high dosed, lisinopril, hydroxyzine hydrochloride. Obviously numerous drugs, insufficiently documented.	[9]
Case 5	Fluticasone/salmeterol 1 puff twice daily. Hydromorphone I mg injection as needed for pain.	-
Case 6	No raw data available.	-
Case 7	Previous drug use as documented in the medical and insurance files included baclophen, diclofenac, acetaminophen, Tylenol, Xanax, Benadryl, cephalexin, but no use of new medications was reported at admission.	-
Case 8	Naproxen, ibuprofen, ritalin, hydrocodone-acetaminophen, diazepam, claritin, and omeprazole. Documented allergies by aspirin, celebrex, celeXa, doxycycline, NSAIDs, and penicillins.	[6]

NSAID: non-steroidal antiinflammatory drug.

**Table 7:** Documented hepatitis serologies of the QMC patients (cases 1—8).

Data	Case 1	Case 2	Case 3	Case 4	Case 5	Case 6	Case 7	Case 8
HAV	Anti-HAV IgM negative.	Anti-HAV IgM negative.	Negative result for anti-HAV total.	Anti-HAV total and IgM negative.	Reported as excluded, but no details provided.	n.a.	Anti-HAV IgM negative and total positive.	Anti-HAV IgM negative.
HBV	Anti-HBs positive, HBV vaccination documented.	Excluded.	Positive tests for anti-HBc and anti-HBs: later stage of acute HBV infection.	Incompletely excluded, missing PCR data.	Reported as excluded, but no details provided.	n.a.	Anti-HBc IgM negative. HBs antigen negative. Anti HBs negative and later positive. Not documented: immunization.	Anti-HBc negative; HBs Ag negative; Anti-HBs positive; no immunization (patient’s refusal).
HCV	Excluded.	Excluded.	Excluded.	Incompletely excluded, missing PCR data.	Reported as excluded, but no details provided.	n.a.	Excluded.	Anti-HCV negative; HCV PCR n.a.
HEV	Anti-HEV IgM negative; anti-HEV-IgG and HEV PCR n.a.	Anti-HEV IgM and IgG negative.	Anti-HEV IgM negative reported at a single time point late in the clinical course. Anti-HEV IgG and HEV PCR n.a.	n.a.	n.a.	n.a.	n.a.	n.a.
CMV	Anti-CMV IgG positive without assessed titer changes in the further course; anti-CMV negative.	Excluded.	Excluded.	Incompletely diagnosed, anti-CMV IgG with high titer, lacking titer change follow up.	Reported as excluded, but no details provided.	Anti-CMV IgG positive.	Anti-CMV IgM negative; CMV DNA <200	Anti-CMV IgM negative.
EBV	Excluded.	Anti-EBV IgG with high titer, subsequent titer changes not evaluated, anti-EBV IgM negative.	Excluded.	Monoscreen negative.	n.a.	n.a.	Excluded.	Anti-EBV IgM negative, anti-EBV IgG positive but no follow up of titer change.
HSV	Excluded.	Excluded.	Excluded.	Not done.	n.a.	n.a.	Excluded.	n.a.
VZV	Anti-VZV IgG positive without assessed titer changes in the further course; anti–CMV IgM negative.	Excluded.	Anti-VZV IgM and IgG, both positive.	Not done.	n.a.	n.a.	Anti-VZV IgM n.a., anti-VZV IgG positive without assessed titer changes in the further course.	n.a.

CMV: cytomegalovirus; EBV: Epstein Barr virus; HAV: hepatitis A virus; HBc: hepatitis B core; HBs: hepatitis B surface; HBV: hepatitis B virus; HCV: hepatitis C virus; HEV: hepatitis E virus; HSV: herpes simplex virus; n.a.: not assessed/ not available; PCR: polymerase chain reaction; VZV: varicella zoster virus.

**Table 8:** Confounders of publications reporting on the QMC patients (cases 1–8).

Report	Major Confounding Variables Presented as Examples	Additional References
CDC, 2013 [3]	1. Type of incriminated OEP product is not identified and confounds assessments.	[6,9]
	2. Lacking details of patients’ interviews, and questionnaires and their analyses.	
	3. Vague case definition, not in line with mainstream clinical hepatology.	
	4. Unclear how negative evaluation of infections including viral hepatitis was achieved.	
	5. Lacking specific and valid exclusion of HEV infection.	
	6. Unclear how preexisting autoimmune hepatitis, chronic alcohol use, and chronic liver diseases were excluded.	
	7. Concomitant use of multiple DS’s as confounders and lacking causality assessment for each used DS.	
	8. Missing reported use of a liver-specific causality assessment such as RUCAM.	
	9. Patients were declared as previously healthy, ignoring documented PMH of significant diseases.	
NEJM, 2014 [4]	1. Upgrading of FDA case numbers by adding non-liver cases to liver cases is a problematic approach and confounds the Perspective article of the NEJM.	[6,9]
	2. Assumed liver cases were not stratified for a specific OEP product.	
	3. The claimed case cluster remained unverified since criterion of one product causing one disease was not satisfied. Patients used two different types of OEP products, many different DS’s, and many different drugs, which confound the cluster concept. This is also not supported as patients experienced not a single type of liver disease but many different ones.	
QMC, 2014 [5]	1. Biased case evaluations, triggered by initial unsubstantiated claims that OEP is hepatotoxic, and premature search for publicity.	[6,9]
	2. Willingly upgrading of causality levels to “probable” causality to let cases appear stronger and suitable for publication, circumventing rejection.	
	3. Case data manipulations invalidate conclusions of the publication and biased case assessment by regulators such as CDC, HDOH, and FDA.	
	4. Neither criteria for case inclusion nor stringent diagnostic protocol at the QMC were provided.	
	5. PMH incompletely presented.	
	6. OLT was erroneously described in one patient (case 7) who never was transplanted according to our review of medical files.	
	7. Most patients were polymedicated by synthetic drugs, not reported in the publication.	
	8. The high number of DS’s used by most patients remained unpublished.	
	9. Case 3 was reported as remaining on the transplantation list for over five months without mentioning that this patient had an acute hepatitis by HBV and VZV coinfection (positive anti-HBs, positive anti-HBc, and positive anti-VZV IgM and IgG), not recognized and treated by QMC physicians.	
	10. Lack of a prospective clinical and causality evaluation.	
	11. Failure to establish and evaluate a control group.	
	12. Selective data presentation in the published report with substantial gap between documented case details and published data with suppression of data not supporting initial claims regarding OEP.	
	13. Use of the outdated RUCAM instead of the updated version.	
	14. Significant misconception of RUCAM assessment for challenge and dechallenge criteria.	
	15. Neglect of individual RUCAM assessment patient by patient, DS by DS, drug by drug, herb by herb, and item by item.	
	16. Publication of four different liver histology cross-sections without attribution to specific patients.	
	17. Before evaluation, the pathologists were told that the patient used OEP, which impeded an unbiased evaluation.	
	18. Rarely valid exclusion of Wilson disease.	
	19. Invalid exclusion of HEV in all QMC cases. Lacking HEV PCR use and application of HEV antibody tests, not approved by the FDA.	
HDOH, 2015 [8]	1. Confounders are vague case definitions, outside mainstream clinical hepatology.	[6,9]
	2. Presented illustrative cases 1 and 2 showed poor case data quality and unprofessional analysis with major confounding variables.	
	3. Irritating statements on “probable” cases, not connected with RUCAM scoring, confound and lead to confusion.	
	4. Comorbidity in half of the cases confounds valid case assessments.	
	5. Alcohol consumption as is described as confounding variable but remained unconsidered for case assessment.	
	6. OEP was used together with another DS by 22/36 patients, not considered as confounders in case assessment and not provided with individual causality gradings for OEP and the other DS’s.	
	7. High frequency of concomitant use of hepatotoxic drugs such as acetaminophen or NSAIDs in 31/44 cases.	
	8. Probable causality for unidentified OEP product by RUCAM assessment in only10/36 cases, whereas in the overwhelming majority clinically relevant causality levels were not established.	
	9. For RUCAM assessment, only OEP was considered, not the other DS’s, herbs and drugs used concomitantly.	
	10. Not accounting for any comedication as possible cause of DILI or HILI confounds case assessments.	
FDA, 2015 [7]	1. MedWatch reports are at risk being incomplete and confounded if not submitted by the caring physicians but provided by patients, their family members, or other non-physicians.	[6,9]
	2. MedWatch system confounds assessment of most HILI and DILI cases.	
	3. MedWatch reports commonly provide high case numbers of low quality.	
	4. The FDA publishes hepatotoxicity cases, not considering mainstream case criteria.	
	5. Causality assessment ignores the use of a liver-specific, validated, and quantitative causality assessment algorithm such as RUCAM.	
	6. The FDA avoids presentation of causality gradings for each suspected product and instead relies on high case numbers.	
	7. The FDA claimed having reviewed patients’ records but overlooked many alternative diagnoses documented in the files.	
	8. The FDA seemed to rely on the statements of the QMC physicians and thereby promoted their biased conclusions.	
	9. The FDA did not realize the willingly and unjustifiable upgrading of causality levels by the QMC physicians, which represents not just a misadventure but a scientific fraud.	

CDC: Centers of Disease DILI: drug induced liver injury; DS: dietary supplement; FDA: Food and Drug Administration; HBV: hepatitis B virus; HBs: Hepatitis B surface; HBc hepatitis B core; HDOH: Hawaii Department of Health; HEV: hepatitis E virus; HILI: herb induced liver injury; NEJM: New England Journal of Medicine; OEP: OxyELITE Pro; OLT: orthotopic liver transplantation; PCR: polymerase chain reaction; PMH: past medical history; QMC: Queen’s Medical Center; RUCAM: Roussel Uclaf Causality Assessment Method; VZV: varicella zoster virus.

## Abbreviations

AAP	acetaminophen
ALF	acute liver failure
HAV	hepatitis A virus
HBV	hepatitis B virus
HBc	hepatitis B core
HBs	hepatitis B surface
HCV	hepatitis C virus
HDOH	Hawaii Department of Health
HDS	herbal dietary supplement
HEV	hepatitis E virus
HILI	herb induced liver injury
FDA	Food and Drug Administration in Washington DC, USA
HAV	hepatitis A virus
HBV	hepatitis B virus
HBc	hepatitis B core
HBs	hepatitis B surface
HCV	hepatitis C virus
HDOH	Hawaii Department of Health
HDS	herbal dietary supplement
HEV	hepatitis E virus
HILI	herb induced liver injury
HSV	herpes simplex virus
LTs	liver tests
N	normal range as multiple of its upper limit
NAFLD	non-alcoholic fatty liver disease
NSAID	non-steroidal antiinflammatory drugs
OC	Oxy Cleanse
OEP	OxyELITE Pro
OLT	orthotopic liver transplantation
OTC	over the counter
PCP	primary care provider
PCR	polymerase chain reaction
PMH	past medical history
QMC	Queen’s Medical Center in Honolulu
RUCAM	Roussel Uclaf Causality Assessment Method
VZV	varicella zoster virus

## References

[1] Sanico M., Soria R. Legends of the Pali. http://www.abstracthawaii.com/journal/legends-of-the-pali.

[2] Chiles W.P. (1995). The Secrets and Mysteries of Hawaii.

[3] CDC, Centers for Disease Control and Prevention (2013). Acute hepatitis and liver failure following use of a dietary supplement intended for weight loss or muscle building—May–October 2013. MMWR Morb Mortal Wkly. Rep..

[4] Cohen P.A. (2014). Hazards of hindsight—Monitoring the safety of nutritional supplements. N. Engl. J. Med..

[5] Roytman M.M., Pörzgen P., Lee C.L., Huddleston L., Kuo T.T., Bryant-Greenwood P., Wong L.L., Tsai N. (2014). Letter to the Editor: Outbreak of severe hepatitis linked to weight-loss supplement OxyELITE Pro. Am. J. Gastroenterol..

[6] Teschke R., Schulze J., Eickhoff A., Wolff A., Frenzel C. (2015). Mysterious Hawaii liver disease case—Naproxen overdose as cause rather than OxyELITE Pro?. J. Liver Clin. Res..

[7] Klontz K.C., DeBeck H.J., LeBlanc P., Mogen K.M., Wolpert B.J., Sabo J.L., Salter M., Seelman S.L., Lance S.E., Monahan C. (2015). The role of adverse event reporting in the FDA response to multistate outbreak of liver disease associated with a dietary supplement. Public Health Rep..

[8] Johnston D.I., Chang A., Viray M., Chatham-Stephens K., He H., Taylor E., Wong L.L., Schier J., Martin C., Fabricant D. (2015). Hepatotoxicity associated with the dietary supplement OxyELITE Pro ^TM^—Hawaii 2013. Drug Test Anal..

[9] Teschke R., Schwarzenboeck A., Frenzel C., Schulze J., Eickhoff A., Wolff A. (2016). The mystery of the Hawaii liver disease cluster in summer 2013: A pragmatic and clinical approach to solve the problem. Ann. Hepatol..

[10] U.S. Department of Health and Human Services. Agency for Toxic Substances and Disease Registry, Division of Toxicology and Environmental Medicine Disease clusters: An overview. http://www.atsdr.cdc.gov/HEC/CSEM/cluster/docs/clusters.pdf.

[11] CDC, Centers for Disease Control and Prevention (1990). Guidelines for investing clusters of health events. MMWR.

[12] Fares A. (2012). Seasonable of viral hepatitis. J. Acad. Med. Sci..

[13] Nainan O.V., Xia G., Vaughan G., Harold S., Margolis H.S. (2006). Diagnosis of hepatitis A virus infection: A molecular approach. Clin. Microbiol. Rev..

[14] Hoofnagle J.H., Nelson K.E., Purcell R.H. (2012). Review article: Hepatitis E. N. Engl. J. Med..

[15] Pavio N., Meng X.J., Renou C. (2010). Zoonotic hepatitis E: Animal reservoirs and emerging risks. Vet. Res..

[16] Fujiwara S., Yokokawa Y., Morino K., Hayasaka K., Kawabata M., Shimizu T. (2014). Chronic hepatitis E: A review of the literature. J. Viral. Hepat..

[17] Li T.C., Chijiwa K., Sera N., Ishibashi T., Etoh Y., Shinohara Y., Kurata Y., Ishida M., Sakamoto S., Takeda N. (2005). Hepatitis E virus transmission from wild boar meat. Emerg. Infect. Dis..

[18] Meng X.J. (2000). Zoonotic and xenozoonotic risks of the hepatitis E virus. Infect. Dis. Rev..

[19] Tei S., Kitajima N., Takahashi K., Mishiro S. (2003). Zoonotic transmission of hepatitis E virus from deer to human beings. Lancet.

[20] WHO: Hepatitis E. http://www.who.int/mediacentre/factsheets/fs280/en/.

[21] Masuda J.I., Yano K., Tamada Y., Takii Y., Ito M., Omagari K., Kohno S. (2005). Acute hepatitis E of a man who consumed wild boar meat prior to the onset of illness in Nagasaki, Japan. Hepatol. Res..

[22] Matsuda H., Okada K., Takahashi K., Mishiro S. (2003). Severe hepatitis E virus infection after ingestion of uncooked liver from a wild boar. J. Infect. Dis..

[23] Kabrane-Lazizi Y., Fine J.B., Elm J., Glass G.E., Higa H., Diwan A., Gibbs C.J., Meng X.J., Emerson S.U., Purcell R.H. (1999). Evidence for widespread infection of wild rats with hepatitis E virus in the United States. Am. J. Trop. Med. Hyg..

[24] Davern T.J., Chalasani N., Fontana R.J., Hayashi P.H., Protiva P., Kleiner D.E., Engle R.E., Nguyen H., Emerson S.U., Purcell R.H. (2011). for the Drug-Induced Liver Injury Network (DILIN). Acute hepatitis E infection accounts for some cases of suspected drug-induced liver injury. Gastroenterology.

[25] Dalton H.R., Fellows H.J., Stableforth W., Josph M., Thurairajah P.H., Warshow U., Hazeldine S., Remnarace R., Ijaz S., Hussaini S.H. (2007). The role of hepatitis E virus testing in drug-induced liver injury. Aliment. Pharmacol. Ther..

[26] Hawaii Department of Health: What is leptospirosis?. http://health.hawaii.gov/about/files/2013/06/leptobrochure.pdf.

[27] Bharti A.R., Nally J.E., Ricaldi J.N., Matthias M.A., Diaz M.M., Lovett M.A., Levett P.N., Gilman R.H., Willig M.R., Gotuzzo E. (2003). Peru-United States Leptospirosis Consortium. Leptospirosis: A zoonotic disease of global importance. Lancet Infect. Dis..

[28] Tandon H.D., Tandon B.N., Ramalingaswami V. (1978). Epidemic of toxic hepatitis in India of possible mycotoxic origin. Arch. Pathol. Lab. Med..

[29] Anfossi L., Calderara M., Baggiani C., Giovannoli C., Arletti E., Giraudi G. (2008). Development and application of solvent-free extraction for the detection of aflatoxin M1 in dairy products by enzyme immunoassay. J. Agric. Food Chem..

[30] Azziz-Baumgartner E., Lindblade K., Gieseker K., Schurz Rogers H., Kieszak S., Njapau H., Schleicher R., McCoy L.F., Misore A., DeCock K. (2005). Case-control study of an acute aflatoxicosis outbreak, Kenya, 2004. Environ/ Health Perspect..

[31] Ferrer Amate C., Unterluggauer H., Fischer R.J., Fernández-Alba A.R., Masselter S. (2010). Development and validation of a LC-MS/MS method for the simultaneous determination of aflatoxins, dyes and pesticides in spices. Anal. Bioanal. Chem..

[32] Garon D., El Kaddoumi A., Carayon A., Amiel C. (2010). FT-IR spectroscopy for rapid differentiation of *Aspergillus flavus, Aspergillus fumigatus, Aspergillus parasiticus* and characterization of aflatoxigenic isolates collected from agricultural environments. Mycopathologica.

[33] Huang B., Han Z., Cai Z., Wu Y., Ren Y. (2010). Simultaneous determination of aflatoxins B1, B2, G1, G2, M1 and M2 in peanuts and their derivative products by ultra-high-performance liquid chromatography-tandem mass spectrometry. Anal. Chim. Acta.

[34] Lupo A., Roebuck C., Dutcher M., Kennedy J., Abouzied M. (2010). Validation study of a rapid ELISA for detection of aflatoxin in corn. Performance Tested Method 050901. AOAC Int..

[35] Ngindu A., Johnson B.K., Ngira J.A., Nandwa H., Jansen A.J., Kaviti J.N., Siongok T.A. (1982). Outbreak of acute hepatitis caused by aflatoxin poisoning in Kenya. Lancet.

[36] Quinto M., Spadaccino G., Palermo C., Centonze D. (2009). Determination of aflatoxins in cereal flours by solid-phase microextraction coupled with liquid chromatography and post-column photochemical derivatization-fluorescence detection. J. Chromatogr. A.

[37] Kakar F., Akbarian Z., Leslie T., Mustafa M.L., Watson J., van Egmond H.P., Omar M.F., Mofleh J. (2010). An outbreak of hepatic veno-occlusive disease in western Afghanistan associated with exposure to wheat flour contaminated with pyrrolizidine alkaloids. J. Toxicol..

[38] Tandon B.N., Tandon H.D., Tandon R.K., Narndranathan M., Joshi Y.K. (1976). An epidemic of veno-occlusive disease of the liver in central India. Lancet.

[39] Tandon H.D., Tandon B., Weston C.F.M., Cooper B.T., Davies J.D., Levine D.F. (1987). Veno-occlusive disease of the liver secondary to ingestion of comfrey. Br. Med. J. (Clin. Res. Ed.).

[40] Tandon R.K., Tandon B.N., Tandon H.D. (1976). Study of an epidemic of venoocclusive disease in India. Gut.

[41] Teschke R., Zhang L., Melzer L., Schulze J., Eickhoff A. (2014). Green tea extract and the risk of drug-induced liver injury. Expert Opin. Drug Metab. Toxicol..

[42] Millonig G., Stadlmann S., Vogel W. (2005). Herbal hepatotoxicity: Acute hepatitis caused by a Noni preparation (*Morinda citrifolia*). Eur. J. Gastroenterol. Hepatol..

[43] Martin A.C., Johnston E., Xing C., Hegeman A.D. (2014). Measuring the chemical and cytotoxic variability of commercially available kava (*Piper methysticum* G. Forster). PLoS ONE.

[44] Schmidt M. (2007). Quality criteria for kava. Herbal. Gram.

[45] Teschke R., Lebot V. (2011). Proposal for a Kava Quality Standardization Code. Food Chem. Toxicol..

[46] Teschke R., Schulze J. (2010). Risk of kava hepatotoxicity and the FDA consumer advisory. J. Am. Med. Assoc..

[47] Teschke R., Sarris J., Schweitzer I. (2012). Kava hepatotoxicity in traditional and modern use: The presumed Pacific kava paradox hypothesis revisited. Br. J. Clin. Pharmacol..

[48] Teschke R., Sarris J., Glass X., Schulze J. (2011). Kava, the anxiolytic herb: Back to basics to prevent liver injury?. Br. J. Clin. Pharmacol..

[49] Watkins P.B. (2015). How to diagnose and exclude drug-induced liver injury. Dig. Dis..

[50] Teschke R., Frenzel C., Wolff A., Eickhoff A., Schulze J. (2014). Drug induced liver injury: Accuracy of diagnosis in published reports. Ann. Hepatol..

[51] Teschke R., Andrade R. (2015). Drug-induced liver injury: Expanding our knowledge by enlarging population analysis with prospective and scoring causality assessment. Gastroenterology.

[52] Teschke R., Frenzel C. (2014). Drug induced liver injury: Do we still need a routine liver biopsy for diagnosis today?. Ann. Hepatol..

[53] Danan G., Teschke R. (2016). RUCAM in drug and herb induced liver injury: The update. Special issue: Drug, herb, and dietary supplement hepatotoxicity. Int. J. Mol. Sci..

[54] Bunchorntavakul C., Reddy K.R. (2013). Review article: Herbal and dietary supplement hepatotoxicity. Aliment Pharmacol. Ther..

[55] Navarro V.J., Barnhart H., Bonkovsky H.L., Davern T., Fontana R.J., Grant L., Reddy K.R., Seeff L.B., Serrano J., Sherker A.H. (2014). Liver injury from herbals and dietary supplements in the U.S. Drug-Induced Liver Injury Network. Hepatology.

[56] Teschke R., Schwarzenboeck A., Eickhoff A., Frenzel C., Wolff A., Schulze J. (2013). Clinical and causality assessment in herbal hepatotoxicity. Expert Opin. Drug Saf..

[57] Teschke R., Eickhoff A. (2015). Herbal hepatotoxicity in traditional and modern medicine: Actual key issues and new encouraging steps. Front. Pharmacol..

[58] Teschke R., Eickhoff A., Schwarzenboeck A., Schmidt-Taenzer W., Genthner A., Frenzel C., Wolff A., Schulze J. (2015). Clinical review: Herbal hepatotoxicity and the call for systematic data documentation of individual cases. J. Liver Clin. Res..

[59] Teschke R., Schulze J., Schwarzenboeck A., Eickhoff A., Frenzel C. (2013). Herbal hepatotoxicity: Suspected cases assessed for alternative causes. Eur. J. Gastroenterol. Hepatol..

[60] Pereira A. Interview about OEP: Doctors at center of liver disease cluster still unsure about cause. http://www.kitv.com/news/hawaii/doctors-at-center-of-liver-disease-cluster-still-unsure-about-cause/22382214.

[61] Huffpost Healthy Living: Aegeline identified as harmful ingredient in OEP. http://www.huffingtonpost.com/2014/01/28/aegeline-oxyelite-pro_n_4683990.html.

[62] Bailey R.L., Gahche J.J., Miller P.E., Thomas P.R., Dwyer J.T. (2013). Why US adults use dietary supplements. JAMA Intern. Med..

[63] Radimer K., Bindewald B., Hughes J., Ervin B., Swanson C., Picciano M.F. (2004). Dietary supplement use by US adults: Data from a National Health and Nutrition examination survey, 1999–2000. Am. J. Epidemiol..

[64] Murphy S.P., Wilkens L.R., Monroe K.R., Steffen A.D., Yonemori K.M., Morimoto Y., Albright C.L. (2011). Dietary supplement use within a multiethnic population as measured by a unique inventory method. J. Am. Diet. Assoc..

[65] Mahady G.B., Low Dog T., Barrett M.L., Chavez M.L., Gardiner P., Ko R., Marles R.J., Pellicore L.S., Giancaspro G.I., Sarma D.N. (2008). United States Pharmacopeia review of the black cohosh case reports of hepatotoxicity. Menopause.

[66] Teschke R., Schmidt-Taenzer W., Wolff A. (2011). Spontaneous reports of assumed herbal hepatotoxicity by black cohosh: Is the liver unspecific Naranjo scale precise enough to ascertain causality?. Pharmacoepidemiol. Drug Saf..

[67] Smilkstein M.J., Knapp G.L., Kulig K.W., Rumack B.H. (1988). Efficacy of oral N-acetylcysteine in the treatment of acetaminophen overdose. N. Engl. J. Med..

[68] Mason A., Sallie R. (1995). What causes fulminant hepatic failure of unknown etiology?. Am. J. Clin. Pathol..

[69] Shakil A.O., Kramer D., Mazariegos G.V., Fung J.J., Rakela J. (2000). Acute liver failure: Clinical features, outcome analysis, and applicability of prognostic criteria. Liver Transpl..

[70] Larson A.M., Polson J., Fontana R.J., Davern T.J., Lalani E., Hynan L.S., Reisch J.S., Schiødt F.V., Ostapowicz G., Shakil A.O. (2005). Acetaminophen-induced acute liver failure: Results of a United States multicentre, prospective study. Hepatology.

[71] Bower W.A., Johns M., Margolis H.S., Williams I.T., Bell B. (2007). Population-based surveillance for acute liver failure. Am. J. Gastroenterol..

[72] Bernal W., Auzinger G., Dhawan A., Wendon J. (2010). Acute liver failure. Lancet.

[73] Reuben A., Koch D.G., Lee W.M., the Acute Liver Failure Study Group (2010). Drug-induced acute liver failure: Results of a U.S. multicenter, prospective study. Hepatology.

[74] Khandelwal N., James L.P., Sanders C., Larson A.M., Lee W.M., the Acute Liver Failure Study Group (2011). Unrecognized acetaminophen toxicity as a cause of indeterminate acute liver failure. Hepatology.

[75] Lee W.M., Larson A.M., Stravitz R.T. AASLD position paper: The management of acute liver failure: Update 2011. https://www.aasld.org/sites/default/files/guideline_documents/alfenhanced.pdf.

[76] Nakayama N., Oketani M., Kawamura Y., Inao M., Nagoshi S., Fujiwara K. (2012). Algorithm to determine the outcome of patients with acute liver failure: A data-mining analysis using decision trees. J. Gastroenterol..

[77] Sgro C., Clinard F., Ouazir K., Chanay H., Allard C., Guilleminet C., Lenoir C., Lemoine A., Hillon P. (2002). Incidence of drug-induced hepatic injuries: A French population-based study. Hepatology.

[78] Teschke R., Stutz G., Strohmeyer G. (1979). Increased paracetamol-induced hepatotoxicity after chronic alcohol consumption. Biochem. Biophys. Res. Commun..

[79] Lok A.S.F., McMahon B.J. (2009). Chronic hepatitis B: Update 2009. Hepatology.

[80] Kamar N., Izopet J., Tripon S., Bismuth M., Hillaire S., Dumortier J., Radenne S., Coilly A., Garrigue V., D’Alteroche L. (2014). Ribavirin for chronic hepatitis E virus infection in transplant recipients. N. Engl. J. Med..

[81] Wedemeyer H., Pischke S., Manns M.P. (2012). Pathogenesis and treatment of hepatitis E virus infection. Gastroenterology.

[82] Ahmed A., Ali I.A., Ghazal H., Fazili J., Nusrat S. (2015). Mystery of hepatitis E virus: Recent advances in its diagnosis and management. Int. J. Hepatol..

[83] Focus Diagnostics: Reference laboratory tests: 2071–2073 Hepatitis E antibodies (IgG, IgM), (IgG), and (IgM). https://www.focusdx.com/focus/1-reference_laboratory/search_frame.asp?f=8.

[84] Zuckerman R.A., Limaye A.P. (2013). Varicella zoster virus (VZV) and herpes simplex virus (HSV) in solid organ transplant patients. Am. J. Transpl..

[85] Luzar B., Ferlan-Marolt V., Poljak M., Sojar V., Stanisavlevic D., Bucovac T., Makoviv S. (2005). Acute fatty liver of pregnancy—An underlying condition of herpes simplex type 2 fulminant hepatitis necessitating liver transplantation. Z. Gastroenterol..

[86] Velasco M., Llamas E., Guijarro-Rojas M., Ruiz-Yague M. (1999). Fulminant herpes hepatitis in a healthy adult. A treatable disease?. J. Clin. Gastroenterol..

[87] Pinna A.D., Rakela J., Demetris A.J., Fung J.J. (2002). Five cases of fulminant hepatitis due to herpes simplex virus in adults. Dig. Dis. Sci..

[88] Rimawi B.H., Meserve J., Rimawi R.H., Min Z., Gnann J.W. (2015). Disseminated herpes simplex virus with fulminant hepatitis. Case Rep. Hepatol..

[89] Teschke R., Schwarzenboeck A. (2009). Suspected hepatotoxicity by cimicifugae racemosae rhizoma (black cohosh, root): Critical analysis and structured causality assessment. Phytomedicine.

[90] Teschke R., Schulze J. (2012). Suspected herbal hepatotoxicity: Requirements for appropriate causality assessment by the US Pharmacopeia. Drug Saf..

[91] Pishvaian A.C., Bahrain M., Lewis J.H. (2006). Fatal varicella-zoster hepatitis presenting with severe abdominal pain: A case report and review of the literature. Dig. Dis. Sci..

[92] Dits H., Frans E., Wilmer A., van Ranst M., Fevery J., Bobbaers H. (1998). Varicella-zoster virus infection associated with acute liver failure. Clin. Infect. Dis..

[93] Anderson D.R., Hunter N.J., Cottrill C., Bissacia E., Klainer A.S. (1994). Varicella hepatitis: A fatal case in a previously healthy immunocompetent adult. Report of a case, autopsy, and review of the literature. Arch. Intern. Med..

[94] Vartian C.V. (1999). Varicella-zoster virus infection associated with acute liver failure. Clin. Infect. Dis..

[95] Ahmed A. (2011). Antiviral treatment of cytomegalovirus infection. Infect. Disord Drug Targets.

[96] Jain M., Duggal S., Chugh T.D. (2011). Cytomegalovirus infection in non-immunosuppressed critically ill patients. J. Infect. Dev. Ctries.

[97] Boeckh M., Geballe A.P. (2011). Cytomegalovirus: Pathogen, paradigm, and puzzle. J. Clin. Investig..

[98] Korman J.D., Volenberg I., Balko J., Webster J., Schiodt F.V., Squires R.H., Fontana R.J., Lee W.M., Schilsky M.I., the Pediatric and Adult Acute Liver Failure Study Groups (2008). Screening for Wilson disease in acute liver failure: A comparison on currently available diagnostic tests. Hepatology.

[99] O’Brien A., Williams R. (2008). Rapid diagnosis of Wilson disease in acute liver failure: No more waiting for the ceruloplasmin level?. Hepatology.

[100] Teschke R., Frenzel C., Schulze J., Eickhoff A. (2013). Herbal hepatotoxicity: Challenges and pitfalls of causality assessment methods. World J. Gastroenterol..

[101] Danan G., Bénichou C. (1993). Causality assessment of adverse reactions to drugs—I. A novel method based on the conclusions of international consensus meetings: Application to drug-induced liver injuries. J. Clin. Epidemiol..

[102] Bénichou C., Danan G., Flahault A. (1993). Causality assessment of adverse reactions to drugs—II. An original model for validation of drug causality assessment methods: Case reports with positive rechallenge. J. Clin. Epidemiol..

[103] Chalasani N., Bonkovsky H.L., Fontana R., Lee W., Stolz A., Talwalkar J., Reddy K.R., Watkins P.B., Navarro V., Barnhart H. (2015). Features and outcomes of 889 patients with drug-induced liver injury: The DILIN Prospective Study. Gastroenterology.

[104] Chen E.Y., Baum K., Collins W., Löve A., Merz M., Olafsson S., Björnsson E.S., Lee W.M. (2012). Hepatitis E masquerading as drug–induced liver injury. Hepatology.

[105] Aithal G.P., Watkins P.B., Andrade R.J., Larrey D., Molokhia M., Takikawa H., Hunt C.M., Wilke R.A., Avigan M., Kaplowitz N. (2011). Case definition and phenotype standardization in drug-induced liver injury. Clin. Pharmacol. Ther..

[106] BfArM (Bundesinstitut für Arzneimittel und Medizinprodukte, Bonn. Federal Institute for Drugs and Medicinal Products in Germany) (2002). Rejection of Drug Risks, Step II. As related to: Kava-Kava (*Piper methysticum*)-containing, and kavain-containing drugs, including homeopathic preparations with a final concentration up to, and including D4. http://www.spc.int/cis/documents/02_0714_BfArM_Kava_Removal.pdf.

[107] Teschke R., Schwarzenboeck A., Hennermann K.H. (2008). Kava hepatotoxicity: A clinical survey and critical analysis of 26 suspected cases. Eur. J. Gastroenterol. Hepatol..

[108] Teschke R. (2010). Kava hepatotoxicity: A clinical review. Ann. Hepatol..

[109] Kuchta K., Schmidt M., Nahrstedt A. (2015). German kava ban lifted by court: The alleged hepatotoxicity of kava (Piper methysticum) of ill- defined herbal drug identity, lacking quality control, and misguided regulatory politics. Planta Med..

[110] Teschke R., Frenzel C., Schulze J., Eickhoff A. (2012). Spontaneous reports of primarily suspected herbal hepatotoxicity by *Pelargonium sidoides*: Was causality adequately ascertained?. Regul. Toxicol. Pharmacol..

[111] Teschke R., Eickhoff A., Wolff A., Frenzel C., Schulze J. (2013). Herbal hepatotoxicity and WHO global introspection method. Ann. Hepatol..

[112] Teschke R., Frenzel C., Wolff A., Herzog J., Glass X., Schulze J., Eickhoff A. (2012). Initially purported hepatotoxicity by *Pelargonium sidoides*: The dilemma of pharmacovigilance and proposals for improvements. Ann. Hepatol..

[113] Teschke R., Glass X., Schulze J. (2011). Herbal hepatotoxicity by Greater Celandine (*Chelidonium majus*): Causality assessment of 22 spontaneous reports. Regul. Toxicol. Pharmacol..

[114] Calitz C., du Plessis L., Gouws C., Steyn D., Steenkamp J., Muller C., Hamman S. (2015). Herbal hepatotoxicity: Current status, examples, and challenges. Expert Opin. Drug Metab. Toxicol..

